# MITF Regulates CFTR Expression to Participate in Myocardial Ischemia–Reperfusion Injury

**DOI:** 10.1002/jgm.70102

**Published:** 2026-08-06

**Authors:** Baoxin Tang, Chenying Zhu, Heqing Wang, Mingkui Gao, Tieyan Li

**Affiliations:** ^1^ Department of Cardiology, Shanghai East Hospital Tongji University School of Medicine Shanghai China; ^2^ Department of Heart Failure, Shanghai East Hospital Tongji University School of Medicine Shanghai China; ^3^ Department of Cardiovascular Surgery, Shanghai East Hospital Tongji University School of Medicine Shanghai China

**Keywords:** arginine biosynthesis, CFTR, MITF, myocardial ischemia–reperfusion injury

## Abstract

**Background:**

Myocardial ischemia–reperfusion injury (MIRI) remains a major clinical problem, and its regulatory mechanisms are not fully defined. This study aimed to identify key MIRI‐related targets and clarify their roles in cellular and animal models.

**Methods:**

Differentially expressed genes in MIRI were screened using the GEO dataset GSE6381, and core genes were identified through protein–protein interaction analysis and weighted gene co‐expression network analysis. External datasets GSE249812 and GSE123342 were used to validate core gene expression. Transcription factors regulating cystic fibrosis transmembrane conductance regulator (CFTR) were predicted using the KnockTF database. In AC16 cells, an oxygen–glucose deprivation/reoxygenation (OGD/OGR) model was established. Microphthalmia‐associated transcription factor (MITF) was knocked down or overexpressed, CFTR was silenced or overexpressed, and cell viability, proliferation, apoptosis, and NO/L‐arginine/citrulline levels were assessed. The MITF‐CFTR interaction was examined by dual‐luciferase assay and chromatin immunoprecipitation‐quantitative PCR (ChIP‐qPCR). A rat ischemia–reperfusion (I/R) model was used to evaluate the effects of MITF silencing and CFTR rescue on cardiac injury.

**Results:**

A total of 42 core genes related to MIRI were identified. CFTR showed consistent upregulation across datasets and was linked to pathways including arginine biosynthesis. CFTR‐related transcription factors included MITF, TP53, and STAT3, but ChIP‐qPCR showed detectable enrichment only for MITF at the CFTR promoter. In AC16 cells, CFTR protein increased during early reoxygenation. OGD/OGR elevated CFTR and MITF, while MITF knockdown or CFTR silencing further reduced cell viability and proliferation, increased apoptosis, and was accompanied by lower NO and citrulline levels and higher L‐arginine levels. MITF overexpression showed the opposite trend, and CFTR overexpression partially reversed the effects of MITF deficiency. In vivo, MITF knockdown aggravated cardiac dysfunction, increased CK and CK‐MB levels, worsened tissue injury, and elevated cleaved caspase‐3 after I/R, whereas CFTR overexpression partly improved these changes.

**Conclusions:**

MITF regulates CFTR expression in MIRI, and disruption of the MITF‐CFTR axis is associated with aggravated injury in cellular and animal models. Restoring CFTR partially mitigates MITF deficiency‐related damage. These findings support the involvement of MITF‐CFTR regulation in MIRI and provide a basis for further mechanistic investigation.

## Graphical abstract

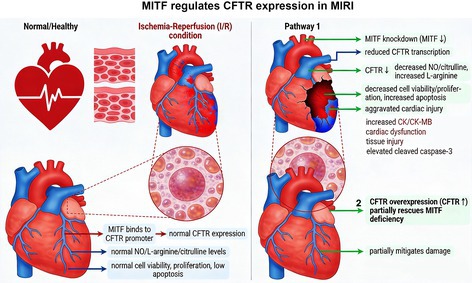

**Abbreviations**
ANOVA: analysis of variance
CCK‐8: Cell Counting Kit 8
DEGs: differentially expressed genes
DO: disease ontology
FBS: fetal bovine serum
FC: fold change
GEO: gene expression omnibus
GO: Gene Ontology
GSEA: gene set enrichment analysis
KEGG: Kyoto Encyclopedia of Genes and Genomes
ME: module eigengene
MIRI: myocardial ischemia–reperfusion injury
NO: nitric oxide
OGD/OGR: oxygen–glucose deprivation/reoxygenation
PPI: protein–protein interaction
ROC: receiver operating characteristic
RT‐qPCR: real‐time quantitative polymerase chain reaction
WGCNA: weighted gene co‐expression network analysis

## 1 Introduction

Myocardial ischemia–reperfusion injury (MIRI) is a common pathological process in patients with acute myocardial infarction following reperfusion therapies such as thrombolysis and interventional treatment [1]. Although reperfusion therapy can restore blood supply to the myocardium, it may also lead to further injury of cardiomyocytes, affecting patient prognosis [2]. Despite the use of antioxidant and anti‐apoptotic interventions in clinical settings, their efficacy is constrained, primarily because the molecular mechanisms underlying MIRI have not been completely clarified [3]. Recent studies have shown the pathogenesis of MIRI involves multilevel regulation, including lipid peroxidation caused by oxidative stress, energy metabolism imbalance due to mitochondrial dysfunction, immune response activation mediated by inflammatory factors (such as TNF‐α and IL‐6), and abnormal activation of apoptosis pathways (such as the caspase family) [4, 5]. These mechanisms are interwoven into a complex regulatory network that still requires in‐depth analysis. Therefore, further exploration of the molecular mechanisms of MIRI and pinpointing effective therapeutic targets holds substantial importance for enhancing patient outcomes.

In the pathological process of MIRI, abnormal regulation of gene expression plays a crucial role [6]. Transcription factors, which bind to gene promoter regions to modulate the transcription of target genes, are crucial in orchestrating biological processes like cell fate determination and stress response [7]. In MIRI, transcription factors dynamically modulate the expression of their downstream target genes by sensing ischemia–reperfusion (I/R) signals, participating in the balance between survival and death of cardiomyocytes [8, 9]. For example, HIF‐1α improved myocardial blood supply by activating angiogenesis‐related genes [10], while NF‐κB exacerbated myocardial injury by promoting the expression of inflammatory factors [11]. However, the specific downstream effectors of many transcription factors in MIRI remain unclear, and the transcriptional regulation of ion transport proteins, particularly chloride channels, has been relatively less explored. Because ion homeostasis affects oxidative stress, mitochondrial function, and apoptotic signaling, disturbances in ion channel expression may be an important but underrecognized contributor to MIRI.

As high‐throughput sequencing technologies have advanced, bioinformatics has emerged as a vital instrument for uncovering the molecular mechanisms underlying complex diseases [12, 13]. Its advantage lies in the ability to integrate multiomics data (such as transcriptomics and proteomics) to efficiently screen for differentially expressed genes (DEGs), construct gene co‐expression networks, and perform pathway enrichment analyses, thereby rapidly identifying core regulatory factors of diseases and overcoming the limitations of traditional experimental methods, which are low‐throughput and high‐cost [14, 15]. In the study of MIRI, bioinformatics has been applied to mine DEGs (such as screening DEGs through gene expression omnibus (GEO) datasets), predict key pathways (such as base excision repair and spliceosome pathways), and construct protein–protein interaction (PPI) networks, providing important clues for subsequent mechanism validation [16]. Recent bioinformatic studies, such as Wu et al., have similarly identified key regulatory genes in MIRI using DEGs, WGCNA, and PPI network analysis [17].

During preliminary transcriptome‐based screening of genes potentially involved in MIRI, several ion transport–associated molecules and their upstream transcriptional regulators emerged as candidates deserving closer examination. This observation is consistent with a growing body of evidence showing that ion channels and transporters influence not only electrophysiological stability but also oxidative stress responses, calcium balance, and cardiomyocyte survival during ischemic challenge. Within this broader context, previous studies have drawn increasing attention to the cystic fibrosis transmembrane conductance regulator (CFTR) and the transcription factor microphthalmia‐associated transcription factor (MITF), both of which have been linked to cellular injury responses and metabolic stress adaptation [18, 19, 20].

CFTR, long regarded for its epithelial chloride channel activity, is now recognized to be expressed in cardiomyocytes, where it participates in chloride handling, redox regulation, and intracellular calcium dynamics [21]. Experimental studies in ischemic preconditioning and postconditioning models have shown that altering CFTR activity can affect myocardial oxidative stress and cell viability, suggesting a context‐dependent contribution of CFTR‐related signaling to the ischemia–reperfusion response [22, 23]. MITF, a basic helix–loop–helix leucine‐zipper transcription factor, regulates genes involved in differentiation, survival, autophagy, and resistance to oxidative injury in several noncardiac damage models, including retinal degeneration and systemic inflammatory stress [24, 25]. Although isolated reports have associated MITF with cardiac hypertrophy and stress signaling [26], its regulatory functions within the heart have not been comprehensively characterized. Bioinformatic predictions further suggest that MITF may regulate CFTR expression. However, whether this transcriptional relationship operates during myocardial ischemia–reperfusion has not been clearly defined. Collectively, these observations suggest that a potential MITF‐CFTR regulatory relationship may be relevant to reperfusion‐related stress responses.

Given these considerations, we aimed to identify regulatory factors that may participate in myocardial ischemia–reperfusion injury. To achieve this, we used an integrated bioinformatic strategy to screen differentially expressed genes, build interaction networks, and predict potential upstream transcriptional regulators. Among the candidates, the MITF‐CFTR axis appeared in preliminary analyses and aligned with existing biological observations, suggesting that it may represent a plausible but understudied regulatory pathway in reperfusion injury. Based on this rationale, the present study investigates whether this transcriptional relationship is involved in cardiomyocyte responses to ischemia–reperfusion stress and seeks to clarify its possible functional relevance.

## 2 Materials and Methods

### 2.1 GEO Transcriptomics Data Analysis in MIRI

Clinical and transcriptomic expression data associated with MIRI were obtained from GEO database (https://www.ncbi.nlm.nih.gov/geo/). GSE123342 dataset includes 67 peripheral blood samples from patients with acute myocardial infarction and 66 peripheral blood samples from patients with MIRI. Clinical data were organized and summarized via R software, and corresponding expression matrices were generated. mRNA expression matrices were extracted using a Perl script, and noncoding RNA expression data were removed. Additionally, a search for “myocardial ischemia reperfusion” was conducted in the GEO database, limited to human samples, resulting in the acquisition of the GSE6381 dataset for external validation. The GSE6381 dataset comprises four right ventricular samples from patients with acute myocardial infarction and four right ventricular tissue samples from patients with MIRI. Transcriptomic and clinical data were downloaded using R packages, and probe names were converted to standard gene names. These data were then combined with the GEO transcriptomic and clinical data for further analysis, including background correction and normalization of transcriptomic data. The disease mRNA expression matrix was merged with previously identified relevant genes. Limma R package was utilized to pinpoint DEGs between disease group and normal control group, with following criteria: *p* value < 0.05 and fold change (FC) ≥ 1.50 (|log_2_FC| ≥ 0.58). This process yielded DEGs related to MIRI. The heatmap package was employed to generate heatmaps and conduct clustering analysis on identified DEGs. *p* value from the differential analysis were transformed using‐log_10_, and ‐log_10_(*p* value) was grouped based on log_2_FC into upregulated DEGs, downregulated DEGs, and DEGs that did not reach statistical significance. Following this, the processed data were imported into R to generate volcano plots. The DEGs were sorted by log_2_FC, and gene set enrichment analysis (GSEA) was carried out.

### 2.2 CFTR Expression Landscape Analysis in MIRI

CFTR expression was extracted and the expression differences in unpaired samples between two groups were compared using ggplot2, stats, and car. R packages. CFTR was selected as the specific gene of interest, and samples were categorized into low‐expression and high‐expression groups according to CFTR expression levels. DESeq2 package was employed to perform single‐gene differential expression analysis on the raw counts matrix of the selected public dataset. The expression of genes identified through single‐gene differential analysis was visualized using the ComplexHeatmap package to generate heatmaps, and correlation expression heatmaps were constructed based on clinical features. Additionally, the expression data of CFTR and all other genes were extracted, and Pearson correlation analysis was performed between CFTR and each of the other genes to identify genes correlated with CFTR. Co‐expression heatmaps and scatter plots of related gene relationships were constructed. Moreover, a PPI network was built for CFTR and its correlated genes.

### 2.3 Clustering of WGCNA

To ensure a consistent analytical workflow, the differentially expressed genes identified from the GSE6381 MIRI microarray dataset were used as the input gene set for WGCNA. From the dataset, genes whose expression levels exceeded the upper quartile of all variances were selected. These genes were subsequently fed into “WGCNA” package within the R software environment to forge a weighted gene co‐expression network tailored for MIRI. A sample clustering dendrogram was crafted, and the optimal soft threshold *β* was ascertained via a scale‐free network topology. Leveraging the *β* exponentiation, an adjacency matrix was meticulously constructed. Thereafter, a topological overlap matrix was formulated to gauge the dissimilarity among genes, which served as the cornerstone for erecting a hierarchical clustering dendrogram. Modules were identified, merged, and visualized as gene dendrograms using a dynamic hybrid cutting method. Following the division into modules, the module eigengene for each module was computed and its association with the clinical characteristics of both MIRI patients and normal individuals was examined. The Pearson correlation coefficient was employed to quantify the relationship between the ME and the clinical traits of the samples. The module exhibiting the strongest correlation with MIRI was designated as the hub module, and a more detailed selection of genes within this module was carried out.

### 2.4 Gene Ontology and KEGG Enrichment Analysis

The core targets of CFTR in MIRI were analyzed using the clusterProfilerGO. R package in R (https://www.r‐project.org/) and Perl. Gene Ontology analysis, which covers cellular component, molecular function, and biological process, was performed to elucidate the functions of these gene products. Additionally, KEGG pathway enrichment analysis was carried out using clusterProfilerKEGG. R package and signaling pathway diagrams were generated with the pathview package. The potential biological functions and signaling pathway mechanisms of CFTR in MIRI treatment were explored by evaluating the enrichment factor values of key pathways.

### 2.5 Relative Expression of Core Targets and Receiver Operating Characteristic Assessment

The intersection analysis of MIRI‐related targets was conducted using R and Perl, and the results were imported into Venny 2.1 (http://bioinfogp.cnb.csic.es/tools/venny/index.html) to create Venn diagrams. MIRI‐related expression matrix data, including transcriptomic files, were obtained from the GEO database. The expression levels of the core targets in MIRI data were analyzed using the ggpubr package to generate box plots. The normalized expression data of MIRI and core targets were combined with clinical data, and receiver operating characteristic (ROC) curves for MIRI core genes were constructed using R packages like survival, caret, glmnet, survminer, and survivalROC. For additional external validation, GSE123342 was processed in the same way, including generation of normalized mRNA expression matrices, comparison of core gene expression between groups, visualization by heatmaps and box plots, and ROC curve analysis.

### 2.6 Immune Infiltration and Immune Function Analysis of MIRI‐Related Core Targets

The CIBERSORT tool was utilized to deconvolute the MIRI expression matrix data, estimating the cellular composition of complex tissues and quantifying the relative abundance of specific cell types based on standardized gene expression data. This process generated an expression matrix for MIRI‐related infiltrating immune cells, which were screened using a Perl script. The limma package in R was used for background correction, normalization, and expression value calculation of the microarray data. The CIBERSORT tool then estimated the immune cell composition in MIRI samples and further analyzed the detailed composition of immune cells in each sample.

### 2.7 Construction of CFTR‐Transcription Factors Regulatory Network and Enrichment Analysis

Transcription factors for core genes and the CFTR gene were identified via KnockTF database (http://www.licpathway.net/KnockTF/index.php). UniProt database (https://www.uniprot.org/) was employed to match and correct target gene names. PPI network between CFTR and transcription factors was constructed using Cytoscape 3.7.2 software (https://cytoscape.org/). Additionally, Disease Ontology (DO)/GO/KEGG enrichment analysis was conducted on potential targets of CFTR transcription factors to elucidate their underlying biological functions.

### 2.8 Cell Culture and Treatment

Human immortalized cardiomyocyte cell line AC16 (CTCC‐003‐0014, MeisenCTCC) was cultured in DMEM/F12 (AC16‐CM, MeisenCTCC) supplemented with 10% fetal bovine serum (FBS) and 1% penicillin/streptomycin. AC16 cells were routinely cultured in a cell incubator at 37°C with 5% CO₂ until they reached the logarithmic growth phase. Cells were allowed to reach approximately 80% confluence before induction of OGD. First, an oxygen–glucose deprivation/reoxygenation (OGD/OGR) model was constructed with following steps: The culture medium in culture flask was replaced with glucose‐free, serum‐free DMEM, and then AC16 cells were placed in an anoxic chamber (95% N₂, 5% CO₂, oxygen concentration < 0.1%) for 3 h. After the anoxic treatment, AC16 cells were quickly removed from the anoxic chamber and the medium was replaced with complete DMEM/F12 containing 10% FBS. AC16 cells were then returned to a normal cell incubator at 37°C with 5% CO₂ for 6 h. To assess early changes in CFTR, AC16 cells subjected to OGD were harvested at 0, 1, 3, and 6 h after reoxygenation, and CFTR protein levels were examined by Western blot. AC16 cells were divided into two groups: control group (AC16 cells were cultured under normal conditions without any special treatment) and OGD/OGR group. This OGD/OGR protocol reflects a delayed reperfusion process that is required to induce myocardial ischemia–reperfusion injury, whereas timely reperfusion generally does not lead to residual myocardial damage.

Furthermore, cells at 50%–60% confluence were used for siRNA transfection. Three types of siRNA were used to knock down MITF. All siRNA transfections were performed at a final concentration of 50 nM. The groups were as follows: NC (AC16 cells transfected with control siRNA), siMITF‐1 (AC16 cells transfected with siRNA targeting MITF‐1), siMITF‐2 (AC16 cells transfected with siRNA targeting MITF‐2), and siMITF‐3 (AC16 cells transfected with siRNA targeting MITF‐3). After identifying the most effective siMITF, further groups were established: NC (AC16 cells transfected with control siRNA), siMITF (AC16 cells transfected with siRNA targeting MITF‐3), NC+OGD/OGR (AC16 cells transfected with control siRNA followed by the construction of OGD/OGR model), and siMITF+OGD/OGR (AC16 cells transfected with siRNA targeting MITF‐3 followed by the construction of the OGD/OGR model).

Additionally, plasmid transfection was performed when the cells reached 70%–80% confluence. To evaluate the effect of MITF overexpression, AC16 cells were transfected with control plasmid (oeNC) or MITF overexpression plasmid (oeMITF). For validation of overexpression efficiency, the cells were divided into two groups: oeNC and oeMITF. For subsequent functional experiments under OGD/OGR conditions, the groups were further divided into oeNC, oeMITF, oeNC+OGD/OGR, and oeMITF+OGD/OGR. Overexpression of CFTR was conducted with the following groups: NC (AC16 cells transfected with control plasmid), NC+OGD/OGR (AC16 cells transfected with control plasmid followed by the construction of the OGD/OGR model), siMITF+OGD/OGR (AC16 cells transfected with siRNA targeting MITF‐3 followed by the construction of the OGD/OGR model), and siMITF+oeCFTR+OGD/OGR (AC16 cells transfected with siRNA targeting MITF‐3 and CFTR overexpression plasmid followed by the construction of the OGD/OGR model). To further evaluate the specific contribution of CFTR, an additional knockdown group was included. AC16 cells were transfected with siRNA targeting CFTR and then subjected to OGD/OGR. The groups for CFTR‐specific analysis were: NC, NC+OGD/OGR, siCFTR+OGD/OGR, siCFTR+OGD/OGR+ARG1 inhibitor (CB‐1158), and siMITF+oeCFTR+OGD/OGR. To examine dynamic changes in CFTR, protein samples were collected at 0, 1, 3, and 6 h after reoxygenation, and CFTR levels were analyzed by Western blot. Transfection was performed according to the instructions of Lipofectamine 2000 (11668‐027, Invitrogen). The siRNA sequences targeting MITF are shown in Table 1. The pCDNA3.1‐CFTR (human)‐3XFLAG (P27726) was synthesized by Wuhan Miaoling Biotechnology Co. Ltd.

**TABLE 1:** The siRNA sequences of MITF.

Names	Sequences (5′→3′)	siRNA sequences
MITF‐human‐1	GACCUAACCUGUACAACAATT	UUGUUGUACAGGUUAGGUCTT
MITF‐human‐2	CGUCCUGUAUGCAGAUGGATT	UCCAUCUGCAUACAGGACGTT
MITF‐human‐3	GGUGCAGACCCACCUCGAATT	UUCGAGGUGGGUCUGCACCTT

### 2.9 Real‐Time Quantitative Polymerase Chain Reaction

CFTR, MITF, STAT3, and TP53 levels in AC16 cells were measured using the real‐time quantitative polymerase chain reaction (RT‐qPCR) method. In the experiment, total RNA was extracted using the TriQuick Reagent Total RNA Extraction Kit (R1100, Solarbio), reverse transcribed into cDNA with the 5×RT SuperMix for qPCR (K1074, APEXbio), and gene expression levels were measured using the 2×SYBR Green qPCR Master Mix (K1070, APEXbio) on the iQ5 system (BIO‐RAD). GAPDH served as the reference gene, and target gene expression levels were calculated using the 2^‐ΔΔCt^ method. Primer sequences are listed in Table 2.

**TABLE 2:** The primers used in this study.

Names	Sequences (5′→3′)	Lengths (bp)
H‐CFTR‐F	AAAAGGCCAGCGTTGTCTCC	211
H‐CFTR‐R	AAACATCGCCGAAGGGCATTA	
H‐MITF‐F	CAGTCCGAATCGGGGATCG	100
H‐MITF‐R	TGCTCTTCAGCGGTTGACTTT	
H‐STAT3‐F	CAGCAGCTTGACACACGGTA	150
H‐STAT3‐R	AAACACCAAAGTGGCATGTGA	
H‐P53‐F	ACCTATGGAAACTACTTCCTGAAA	141
H‐P53‐R	CTGGCATTCTGGGAGCTTCA	
H‐GAPDH‐F	CATCATCCCTGCCTCTACTGG	259
H‐GAPDH‐R	GTGGGTGTCGCTGTTGAAGTC	

### 2.10 Western Blot

Western blot analysis was performed on both AC16 cells and rat heart tissues. Total protein was extracted using RIPA buffer (P0013B, Beyotime) and quantified using the BCA kit (BL521A, Biosharp). Equal amounts of protein (20–30 μg per lane) were loaded onto 15% polyacrylamide gels and separated by SDS‐PAGE at 80 V for stacking and 120 V for resolving. Proteins were transferred to PVDF membranes (ISEQ00010, MerckMillipore) at 100 V for 90 min in ice‐cold transfer buffer. Membranes were blocked with 5% skim milk in TBST for 1 h at room temperature and incubated overnight at 4°C with primary antibodies: CFTR (HA723168, HuaBio; recognizes mature CFTR), MITF (ET1702‐86, HuaBio), Phospho‐STAT3‐Y705 (AP0705, ABCLONAL), STAT3 (ET1607‐38, HuaBio), P53 (60283‐2‐IG, Proteintech), cleaved caspase‐3 (9664, CST), and GAPDH (60004‐1‐Ig, Proteintech) at recommended dilutions (1:1000–1:3000, depending on antibody datasheet). Membranes were subsequently co‐incubated with Goat anti‐Rabbit IgG‐HRP (BL003A, Biosharp) or Goat anti‐Mouse IgG‐HRP (BL001A, Biosharp) for 1 h at room temperature. Proteins were visualized using the ECL chemiluminescent substrate (ultrasensitive) (K‐12045‐D50, Advansta).

### 2.11 Cell Counting Kit 8 Assay

AC16 cell viability was evaluated with a Cell Counting Kit 8 (CCK‐8 kit) (CA1210, Solarbio). Logarithmic phase cells were harvested, washed with PBS, and digested into a single‐cell suspension using 0.25% trypsin (25200‐072, Gibco). The digestion was stopped with complete medium, and cells were resuspended and adjusted to a concentration of 3 × 10^4^ cells/mL. Then, 100 μL (3 × 10^3^ cells) of the suspension was seeded into each well of a 96‐well plate. Each cell line was tested in triplicate daily. The cells were cultured in a 37°C, 5% CO₂ incubator with 100 μL of complete medium per well. Cells were transfected for 24 h and then subjected to OGD/OGR, and viability was assessed immediately after reoxygenation when the confluency was roughly 50%–80%. After incubation, 10 μL of CCK‐8 solution was added to each well, followed by a 2‐h incubation at 37°C. Absorbance at 450 nm was assessed with a microplate reader.

### 2.12 EdU Assay

AC16 cell proliferation was assessed with BeyoClick EdU‐488 kit (C0071S, Beyotime). Log‐phase AC16 cells were harvested, medium was replaced with an equal volume of 2 × EdU working solution (20 μM) to reach a final concentration of 10 μM, and incubated for 2 h. Cells were then fixed with 4% paraformaldehyde and permeabilized with 0.3% Triton X‐100 in PBS for 10–15 min. Click reaction solution (50 μL) was added to each well and incubated in the dark for 30 min, followed by three washes. EdU labeling was carried out after 24 h of siRNA or plasmid transfection and completion of the OGD/OGR procedure, and images were obtained immediately after reoxygenation with cell confluency maintained at approximately 50%–80%. Cells were stained with 1× Hoechst 33342 for 10 min in the dark and washed three times. Finally, AC16 cells were observed and imaged using a fluorescence microscope.

### 2.13 Flow Cytometry

Apoptosis in AC16 cells was assessed via flow cytometry using the Annexin V‐FITC Apoptosis Detection Kit (C1062S, Beyotime). Culture medium was carefully removed, and cells were gently digested with EDTA‐free trypsin. Digestion was stopped by adding serum‐containing medium, and cells were pipetted to form a single‐cell suspension. Cells were collected by centrifugation at 300 g for 5 min at 4°C, washed twice with cold PBS, and resuspended in 195 μL of 1× Binding Buffer to a concentration of 1–5 × 10^5^ cells/mL. Annexin V‐FITC (5 μL) and PI staining solution (10 μL) were added, and the mixture was gently mixed and incubated in the dark at room temperature for 10–20 min, with gentle inversion to enhance staining. Cells were transfected for 24 h, exposed to OGD/OGR, and stained immediately after reoxygenation. At the time of flow cytometry, cell confluency was between 50% and 80%. Cells were then resuspended in 400 μL of 1 × Binding Buffer and analyzed by flow cytometry. Annexin V‐FITC fluorescence was detected in the FL1 channel (excitation 488 nm and emission 525 nm), and PI fluorescence in the FL2 or FL3 channel (excitation 535 nm and emission 617 nm).

### 2.14 Dual‐Luciferase Reporter Gene Assay

Bioinformatics methods were utilized to forecast the interaction sites between CFTR and MITF. The interaction was confirmed through a dual‐luciferase reporter gene assay, conducted using the Luc‐Pair Duo‐Luciferase Assay Kit 2.0 (# LF001, iGeneBio) as per the manufacturer's protocol. Wild‐type (wt) luciferase reporter plasmids (CFTR‐promoter‐wt) and mutant (mut) plasmids (CFTR‐promoter‐mut) targeting the binding sites of CFTR mRNA 3′UTR and MITF were designed and synthesized. AC16 cells were cultured and divided into the following groups: CFTR‐promoter‐wt+NC group (AC16 cells co‐transfected with oeNC and CFTR‐promoter‐wt), CFTR‐promoter‐wt+oeMITF group (AC16 cells co‐transfected with oeMITF and CFTR‐promoter‐wt), CFTR‐promoter‐mut‐1/2+NC group (AC16 cells co‐transfected with oeNC and CFTR‐promoter‐mut‐1/2), and CFTR‐promoter‐mut‐1/2+oeMITF group (AC16 cells co‐transfected with oeMITF and CFTR‐promoter‐mut‐1/2). After 48 h of transfection, the dual‐luciferase activity was detected. pCMV‐Myc‐MITF (human) (P22379) was provided by Wuhan Miaoling Biotechnology Co. Ltd., and CFTR‐promoter‐wt and CFTR‐promoter‐mut were purchased from GENCEFE Biotech.

### 2.15 Chromatin Immunoprecipitation‐qPCR

Chromatin immunoprecipitation‐qPCR (ChIP‐qPCR) was carried out to determine whether MITF binds to the CFTR promoter in AC16 cells. Cells were fixed with 1% formaldehyde for 10 min at room temperature, and the reaction was stopped with 0.125 mol/L glycine. After washing with cold PBS, nuclei were collected and chromatin was sheared by sonication to fragments of approximately 200–500 bp. The supernatant was incubated overnight at 4°C with anti‐MITF antibody (ET1702‐86, HuaBio) or normal rabbit IgG (BL003A, Biosharp), followed by protein A/G agarose bead enrichment. After sequential washing, the bound chromatin was eluted and the cross‐links were reversed at 65°C. DNA was purified and subjected to qPCR using primers targeting the predicted MITF‐responsive regions within the CFTR promoter. Region 1: FP: TTCCTTGGTCAGCTTCTATG, RP: TCTTTCCTGGTGTCTAACT; Region 2: FP: GAAGGGCAAAGCAGAGCTAT, RP: CAAACACAATGTATGCTTGC. Relative enrichment for each region was calculated against input DNA and normalized to IgG control.

### 2.16 Animal

All animal procedures in this study were carried out in accordance with institutional guidelines for the care and use of laboratory animals. The experimental protocol was reviewed and approved by the Guangzhou Forevergen Biosciences Animal Experimentation Ethics Committee (approval number: IACUC‐AEWC‐F250912002). All efforts were made to minimize discomfort during surgery and postoperative monitoring. Male Sprague–Dawley rats (350–400 g) were housed under controlled temperature and humidity with free access to food and water. Before surgery, animals were acclimated for at least 1 week. Adequate anesthesia and analgesia were used throughout the I/R procedure to reduce pain and stress. At the end of the experiment, animals were euthanized under deep anesthesia according to institutional guidelines.

### 2.17 Rat Myocardial I/R Model

Rats were randomly assigned to four groups (*n* = 3/group): sham, I/R, shMITF+I/R, and shMITF+oeCFTR+I/R. Myocardial I/R was induced by left anterior descending coronary artery ligation for 30 min followed by reperfusion. In the sham group, the suture was placed under the artery but not tightened. All procedures followed institutional ethical guidelines. This protocol creates a brief period of ischemia before reperfusion, which models delayed restoration of blood flow—the key condition that leads to myocardial ischemia–reperfusion injury, rather than the harmless outcome seen with immediate reperfusion.

### 2.18 AAV‐MITF/AAV‐CFTR Administration In Vivo

To modulate MITF and CFTR expression in rats, AAV9‐shMITF and AAV9‐CFTR were used. According to the manufacturer's recommended dose, vectors were injected via the tail vein 4 weeks before I/R surgery, ensuring stable expression at the time of modeling. The shMITF+I/R group received AAV‐shMITF alone, whereas the shMITF+oeCFTR+I/R group received a combination of AAV‐shMITF and AAV‐CFTR. Control vectors were used for the sham and I/R groups.

### 2.19 ELISA Assay

At the end of reperfusion or after cell culture treatments, blood or culture supernatants were collected and centrifuged to isolate serum or supernatants. Creatine kinase (CK) and creatine kinase‐MB (CK‐MB) levels were measured using commercial ELISA kits (RX300944R and RX301189R, Ruixinbio) according to the manufacturer's instructions. Nitric oxide (NO) levels were quantified using a microplate‐based NO assay kit (bc1475, Solarbio), L‐arginine (L‐Arg) levels were measured using a colorimetric L‐Arg assay kit (E‐BC‐K850‐M, Elabscience), and citrulline (Cit) levels were determined using a Citrulline ELISA kit (CEA505Ge, Cloud‐Clone Corp.), all following the respective manufacturers' protocols.

### 2.20 Echocardiographic Evaluation

Transthoracic echocardiography was performed to evaluate left ventricular systolic function. Rats were lightly anesthetized with isoflurane and positioned supine on a warming platform. M‐mode images were obtained at the level of the papillary muscles using a high‐frequency ultrasound system. Left ventricular end‐diastolic diameter (LVEDD) and end‐systolic diameter (LVESD) were measured from at least three consecutive cardiac cycles. Left ventricular ejection fraction (LVEF) and fractional shortening (LVFS) were calculated automatically by the system software. Functional parameters were compared among the sham, I/R, shMITF+I/R, and shMITF+oeCFTR+I/R groups.

### 2.21 Hematoxylin–Eosin Staining

Left ventricular tissue was collected immediately after functional assessment and rinsed gently in cold saline to remove residual blood. Samples were fixed in 4% paraformaldehyde (BL539A, Biosharp) for at least 24 h, embedded in paraffin, and cut into 4‐μm sections using a rotary microtome. Standard hematoxylin–eosin staining was carried out with the hematoxylin–eosin (H&E) staining kit (G1120, Solarbio) according to the manufacturer's instructions. Sections were sequentially deparaffinized in xylene, rehydrated through graded ethanol, stained with hematoxylin, differentiated, blued, and then counterstained with eosin. After dehydration and mounting, tissue morphology was examined under light microscopy at both low (4 ×) and high (20 ×) magnification. Myocardial fiber arrangement, cytoplasmic changes, inflammatory cell infiltration, and structural injury were compared across the four groups.

### 2.22 Statistical Analysis

All statistical analyses were performed using GraphPad Prism 8.0. Quantitative data are presented as mean ± standard deviation (SD). For comparisons between two groups, a two‐tailed Student's *t*‐test was used. One‐way ANOVA was applied for multiple‐group comparisons, followed by an appropriate post hoc test. Data involving two independent variables were analyzed using two‐way ANOVA followed by Sidak's multiple comparisons test. *p* < 0.05 was considered statistically significant. Unless otherwise stated, all in vitro experiments were performed with three independent biological replicates using cells from different passages, and each biological replicate included three technical replicates where applicable.

## 3 Results

### 3.1 Disease Target Screening for MIRI

**FIGURE 1:**
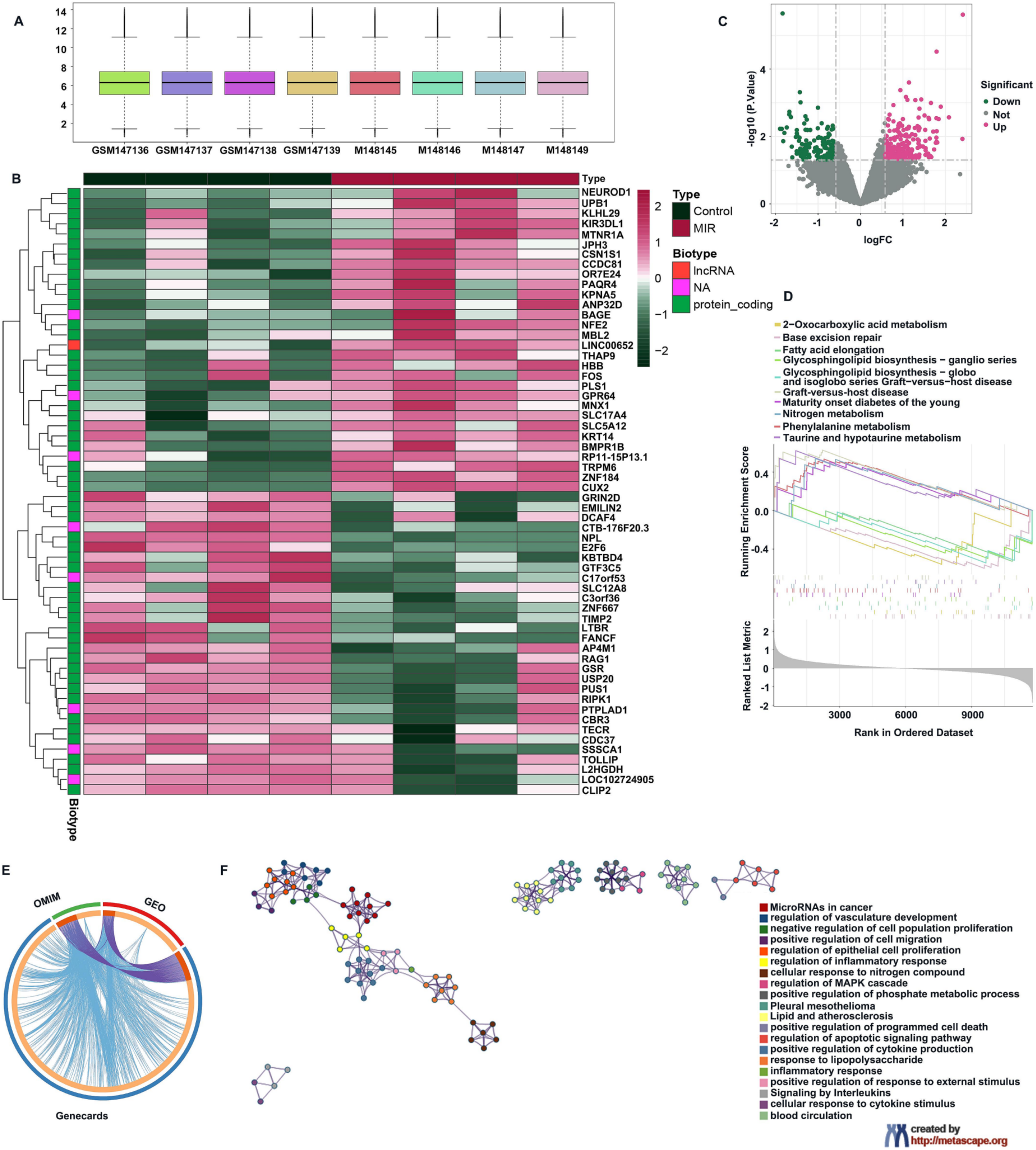
**Disease target screening for MIRI.** (A) Standardized sample distribution of the GSE6381 dataset, showing balanced expression profiles across subjects. (B) Heatmap of DEGs in GSE6381, illustrating distinct transcriptional patterns between groups. (C) Volcano plot highlighting significantly upregulated and downregulated genes. (D) GSEA pathways enriched in the MIRI group. (E) Distribution of MIRI‐related disease targets across database sources. (F) PPI network revealing major interacting proteins associated with MIRI. “Control” refers to myocardial samples from AMI patients without reperfusion injury.

In gene expression studies targeting MIRI, the screening criteria were set as follows: “myocardial ischemia reperfusion” and human. Our study used microarray dataset GSE6381 (comprising four right ventricular samples from patients with acute myocardial infarction and four right ventricular tissue samples from patients with MIRI). After background correction and normalization, Figure 1A displayed the sample distribution of the GSE6381 dataset. According to the criteria of *p* value < 0.05 (adjusted where applicable) and FC ≥ 1.50 (|log2FC| ≥ 0.58), 215 upregulated and 151 downregulated DEGs were identified in the GSE6381 dataset. Heatmaps were generated for the DEGs (Figure 1B), showing the expression patterns of DEGs across various samples. The processed data were imported into R to create a volcano plot (Figure 1C), which illustrated the distribution of DEGs, with significantly upregulated and downregulated genes marked in pink and green, respectively. After sorting the MIRI genes by log2FC, GSEA revealed significant enrichment of the GSE6381 dataset in the top 10 pathways, including 2‐oxocarboxylic acid metabolism, base excision repair, fatty acid elongation, glycosphingolipid biosynthesis—ganglio series, glycosphingolipid biosynthesis—globo and isoglobo series, graft‐versus‐host disease, maturity onset diabetes of the young, nitrogen metabolism, phenylalanine metabolism, and taurine and hypotaurine metabolism, compared to control (myocardial samples from AMI patients without reperfusion injury) (Figure 1D; NES > 0, FDR < 0.25). By searching “myocardial ischemia reperfusion” in the GeneCards, OMIM, and GEO databases, 1761, 192, and 366 targets were obtained, respectively. After merging and removing duplicates, 2183 MIRI‐related targets were identified. The distribution of these disease targets was shown using Metascape (Figure 1E), and a PPI network of MIRI‐related targets was constructed using Metascape (Figure 1F).

To further validate the expression of seven core genes, we analyzed an independent external dataset (GSE123342), which included 67 peripheral blood samples from acute myocardial infarction patients and 66 samples from myocardial ischemia–reperfusion patients. The results showed that CFTR, PSMC3, AHNAK, and CEMP1 were upregulated in ischemia–reperfusion samples, whereas ARG1 was downregulated. Their expression patterns are visualized as a heatmap and boxplots in Figure S1A,B. ROC curve analysis indicated that CFTR, PSMC3, AHNAK, and ARG1 exhibited an AUC greater than 0.60, suggesting potential diagnostic value, while CEMP1 showed a trend toward upregulation with an AUC of 0.579 (Figure S1C).

### 3.2 WGCNA of Core Genes

**FIGURE 2:**
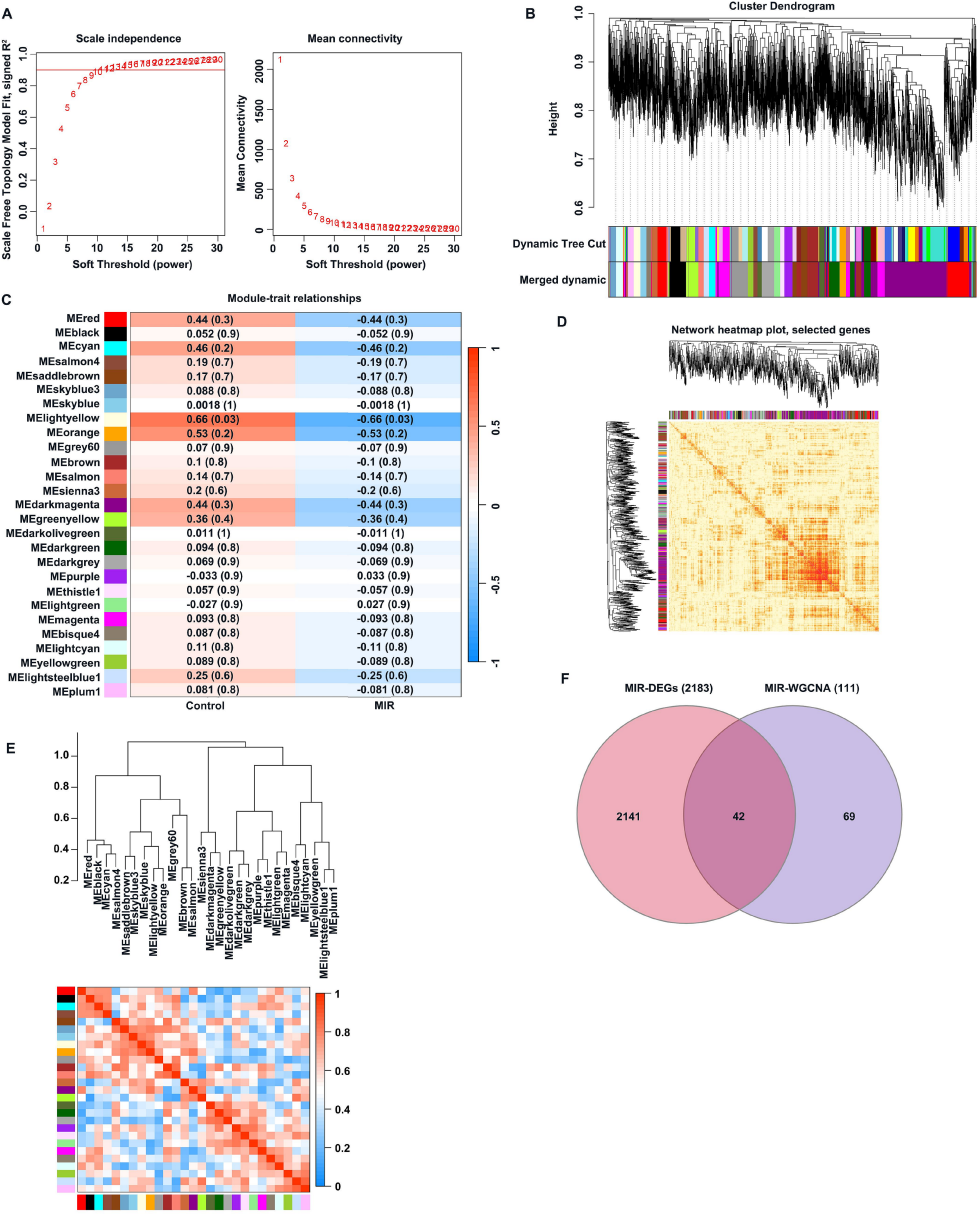
**WGCNA of core genes.** (A) Determination of soft‐threshold power for scale‐free topology (*β* = 9). (B) Clustering analysis showing no outlier samples. (C) Gene dendrogram identifying co‐expression modules. (D) Module‐trait correlations, with the light‐yellow module most strongly associated with MIRI. (E) Heatmap displaying module‐specific expression patterns. (F) Intersection of WGCNA‐derived genes and disease‐related targets, yielding 42 core genes. Analyses were performed on eight samples in the GSE6381 dataset.

The expression matrix of DEGs related to MIRI in dataset was subjected to WGCNA. For the GSE6381 dataset, the value of the soft threshold (*β*) was chosen to be 9, at which the correlation coefficient first exceeded 0.9 (Figure 2A). This result indicated that the dataset was suitable for further construction of a gene co‐expression network. Correlation coefficients were calculated based on the expression levels of the eight samples, followed by hierarchical clustering analysis to detect any significant outliers. The results indicated that the eight samples clustered well, with no obvious outliers, making them suitable for subsequent data analysis (Figure 2B). A gene co‐expression network was established using a soft threshold (*β*) of 9. The dissimilarity coefficients of the DEGs were determined, and modules were delineated with the hybrid dynamic tree cutting method, ultimately dividing all genes into 27 modules (Figure 2C). Further correlation analysis was performed between the 27 gene co‐expression modules and the MIRI microarray data. The results showed that one co‐expression module (the light‐yellow module) was significantly associated with MIRI (Figure 2D), with a correlation coefficient |*r*| ≥ 0.60 and *p* value < 0.05, indicating that the light‐yellow module may contain hub genes related to MIRI, yielding a total of 111 hub genes. Finally, a heatmap was generated to illustrate the expression variations of the genes derived from WGCNA across distinct clusters (Figure 2E). A Venn diagram was employed to examine the overlap between the 2183 MIR‐related genes obtained earlier and the 111 hub genes identified by WGCNA, ultimately resulting in 42 core genes for MIR (Figure 2F).

### 3.3 External Dataset Validation and Enrichment Analysis

**FIGURE 3:**
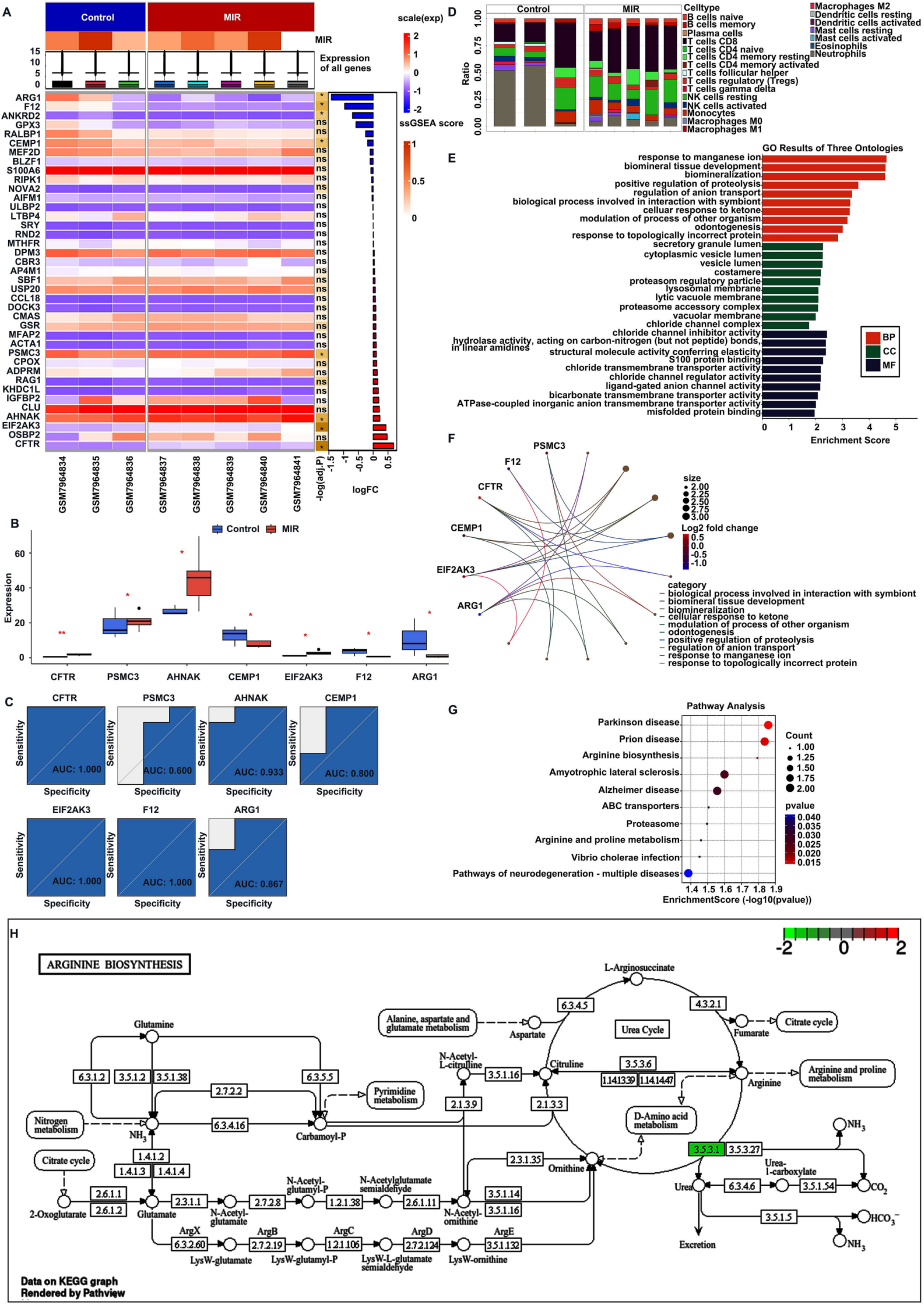
**External dataset validation and enrichment analysis.** (A,B) Core gene expression patterns across samples and corresponding box plots. (C) ROC curves demonstrating diagnostic value of several core genes. (D) Immune infiltration profiles comparing MIRI and control samples. (E,F) GO functional enrichment showing involvement in ion transport and cellular structure. (G) KEGG pathway enrichment featuring arginine biosynthesis and related processes. (H) Mapping of identified genes onto the arginine biosynthesis pathway.

The relative expression levels of intersecting genes were analyzed by ggpubr package and visualized with heatmaps and box plots. Figure 3A,B showed that PSMC3, AHNAK, EIF2AK3, and CFTR gene levels were significantly upregulated in MIRI, while ARG1, F12, and CEMP1 were notably downregulated (*p* < 0.05). ROC curve analysis was conducted on these genes to evaluate their sensitivity and specificity in diagnosing MIRI. In GSE249812 dataset, the area under curve values for ARG1, F12, CEMP1, PSMC3, AHNAK, EIF2AK3, and CFTR all exceeded 0.60, suggesting significant diagnostic value (Figure 3C). The expression matrix data of MIRI were analyzed by CIBERSORT package to estimate the composition of immune cells in MIRI and control (myocardial samples from AMI patients without reperfusion injury), and the results were presented in bar charts (Figure 3D). Finally, Gene Ontology and KEGG pathway enrichment analyses were performed on seven potential core target genes of MIRI utilizing Bioconductor and clusterProfiler packages in R. Gene Ontology analysis revealed that the biological processes were mainly enriched in response to manganese ion, biomineral tissue development, and biomineralization; the cellular components were primarily enriched in secretory granule lumen, cytoplasmic vesicle lumen, and vesicle lumen; and the molecular functions were mainly enriched in chloride channel inhibitor activity, hydrolase activity acting on carbon‐nitrogen (but not peptide) bonds in linear amidines, and structural molecule activity conferring elasticity (Figure 3E,F). The KEGG pathway enrichment analysis indicated that these genes were mainly involved in pathways related to Parkinson disease, prion disease, arginine biosynthesis, amyotrophic lateral sclerosis, Alzheimer disease, and ABC transporters, etc. (Figure 3G). Figure 3H further illustrated the Arginine biosynthesis signaling pathway. The comprehensive analysis indicated that the core genes showed enrichment signals in the arginine biosynthesis pathway, suggesting a possible association that warrants further investigation. These findings led us to focus on CFTR as a representative core gene and to explore its upstream transcriptional regulation in more detail.

### 3.4 Construction of CFTR‐Transcription Factor Regulatory Network and Validation of Expression Levels

**FIGURE 4:**
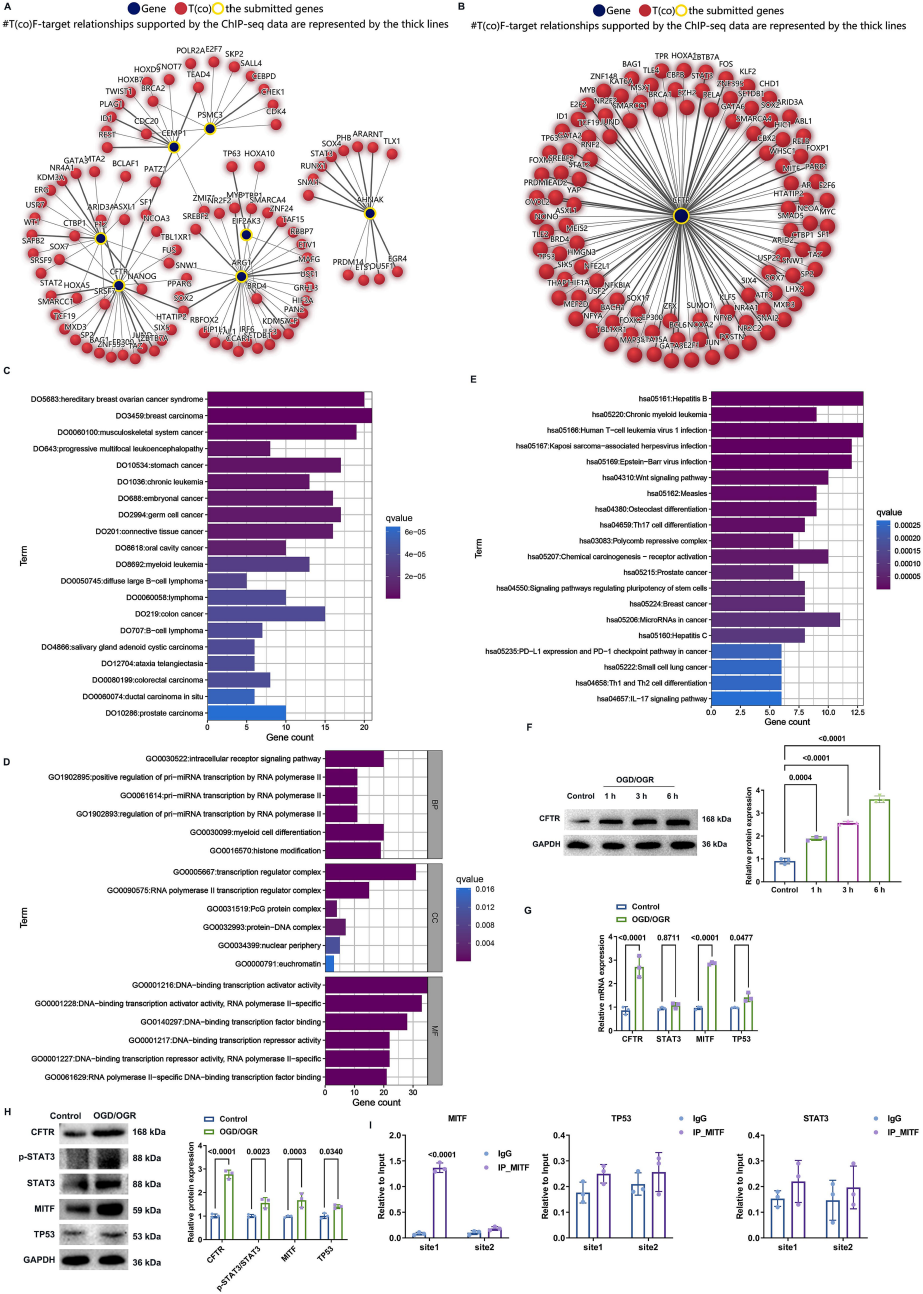
**CFTR‐transcription factor regulatory network and expression validation.** (A) Regulatory network of transcription factors associated with the seven core genes. (B) CFTR‐centered transcription factor network showing MITF, TP53, and STAT3. (C) DO enrichment analysis of CFTR‐related transcription factor targets. (D) GO enrichment analysis of CFTR‐related transcription factor targets. (E) KEGG pathway enrichment analysis of CFTR‐related transcription factor targets. F. CFTR protein expression in AC16 cells at 1, 3, and 6 h after reoxygenation following OGD, with the untreated control shown for comparison. (G) mRNA expression of CFTR, STAT3, MITF, and TP53 in control and OGD/OGR‐treated AC16 cells. (H) Protein expression of CFTR, p‐STAT3/STAT3, MITF, and TP53 in control and OGD/OGR‐treated AC16 cells. I. ChIP‐qPCR analysis of MITF, TP53, and STAT3 at two predicted regions of the CFTR promoter in AC16 cells. For panels F–I, all cell experiments were performed with three biological replicates. Data are presented as mean ± SD and analyzed by one‐way ANOVA or two‐way ANOVA (I).

The seven core genes identified above were input into the KnockTF database to retrieve the 101 transcription factors with the highest relevance to these core genes, and a core gene‐transcription factor regulatory PPI network was constructed (Figure 4A). Further analysis of the core gene CFTR revealed its closely related transcription factors, including TP53, STAT3, and MITF, with the PPI network shown in Figure 4B. Subsequently, DO/GO/KEGG enrichment analysis was performed on the potential targets of CFTR transcription factors. The DO analysis showed that four transcription factors associated with CFTR (HIF1A, POSTN, SOX2, and TP53) were annotated to disease terms related to acute myocardial infarction (Figure 4C). These genes have been reported in settings involving ischemic injury, extracellular matrix changes, or stress responses, which is consistent with their potential involvement in MIRI. GO analysis further indicated that these genes were enriched in intracellular receptor signaling and in processes regulating pri‐miRNA transcription (Figure 4D). These functions fit with their roles as nuclear regulators that respond to cellular stress signals. In the KEGG analysis, the enriched pathways included several inflammation‐related or proliferation‐associated signaling pathways (Figure 4E). Although these pathways are not specific to cardiac tissue, they reflect broader transcriptional programs that may also be activated during ischemia–reperfusion injury. Together, these analyses suggested that CFTR‐related transcription factors participate in stress‐responsive regulatory networks relevant to MIRI, which formed the basis for our subsequent validation experiments. Among these transcription factors, MITF showed the strongest association with CFTR and contained predicted binding motifs within the CFTR promoter, so it was selected for further experimental validation.

To further examine CFTR changes during early reoxygenation, we measured its protein levels at 0, 1, 3, and 6 h. CFTR levels were low at the beginning of reoxygenation and appeared higher at 3 and 6 h (Figure 4F). We then measured transcriptional changes. Compared with the control group, CFTR, MITF, and TP53 mRNA levels were significantly increased, whereas STAT3 mRNA showed no obvious change (Figure 4G). At the protein level, CFTR, MITF, and TP53 were also elevated, and the p‐STAT3/STAT3 ratio increased despite stable STAT3 mRNA, suggesting activation of STAT3 mainly through phosphorylation rather than transcriptional upregulation (Figure 4H). To directly examine transcription factor binding to the CFTR promoter, ChIP‐qPCR was performed in AC16 cells for MITF, TP53, and STAT3 using primers targeting two predicted MITF‐responsive regions. Significant enrichment of CFTR promoter sequences was observed only in the MITF‐immunoprecipitated fraction, while no clear enrichment was detected for TP53 or STAT3 (Figure 4I). These results indicate that MITF interacts with the CFTR promoter, whereas TP53 and STAT3 do not show detectable binding.

### 3.5 MITF Knockdown and Overexpression Effects on AC16 Cells Under OGD/OGR Conditions

**FIGURE 5:**
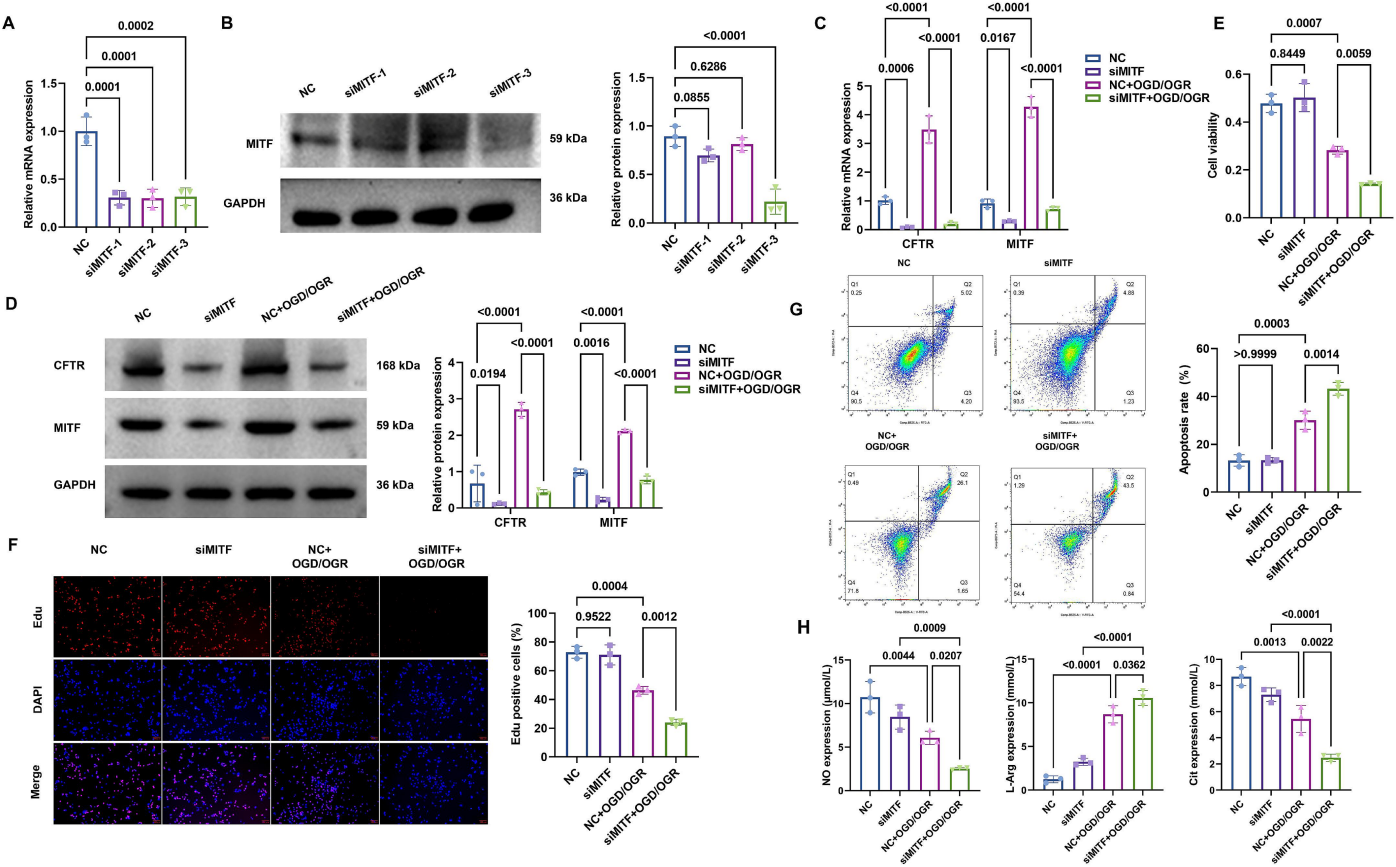
Knockdown of MITF exacerbated cell injury in the OGD/OGR model. (A,B) MITF knockdown efficiency confirmed by RT‐qPCR and Western blot. (C,D) RT‐qPCR and Western blot analysis of MITF and CFTR expression after MITF knockdown and OGD/OGR treatment. The CFTR band shown corresponds to the mature band (C). MITF and CFTR levels were reduced in MITF‐silenced cells under OGD/OGR conditions. (E) Reduced cell viability following MITF knockdown by CCK‐8. (F) Impaired proliferation shown by EdU staining. (G) Increased apoptotic rate detected by flow cytometry. (H) NO, L‐arginine, and citrulline levels in AC16 cells under four conditions: NC, siMITF, NC+OGD/OGR, and siMITF+OGD/OGR. Data are shown as mean ± SD from three biological replicates.

Next, we utilized three types of siRNA to knock down MITF. In comparison with the NC group, MITF mRNA and protein levels were notably reduced in the siMITF‐3 groups, which was used for subsequent experiments (Figure 5A,B). After knocking down MITF and constructing the OGD/OGR model, CFTR and MITF levels were lower in the siMITF+OGD/OGR group compared with NC, whereas both genes were increased in the NC+OGD/OGR group when compared with NC (Figure 5C,D). Functional experiments showed that compared with NC, cell viability and proliferation were decreased, and apoptosis was increased in the NC+OGD/OGR group. These effects were more pronounced in the siMITF+OGD/OGR group (Figure 5E–G). In addition, we measured NO, L‐arginine, and citrulline levels in the same groups. Compared with NC, the OGD/OGR model showed lower NO and citrulline levels and higher L‐arginine levels. In the siMITF+OGD/OGR group, NO and citrulline were further reduced, and L‐arginine was further increased compared with the OGD/OGR group (Figure 5H).

To examine the opposite effect, AC16 cells were transfected with MITF overexpression plasmid. MITF mRNA and protein levels were elevated in the oeMITF group compared with NC (Figure S2A–B). Under OGD/OGR conditions, oeMITF cells showed higher viability and proliferation and lower apoptosis than NC+OGD/OGR (Figure S2C–E). Metabolic measurements indicated higher NO and citrulline levels and lower L‐arginine levels in oeMITF+OGD/OGR cells (Figure S2F).

### 3.6 MITF Knockdown Aggravated OGD/OGR‐Induced Injury in AC16 Cells, Whereas CFTR Overexpression Partially Reversed These Effects

**FIGURE 6:**
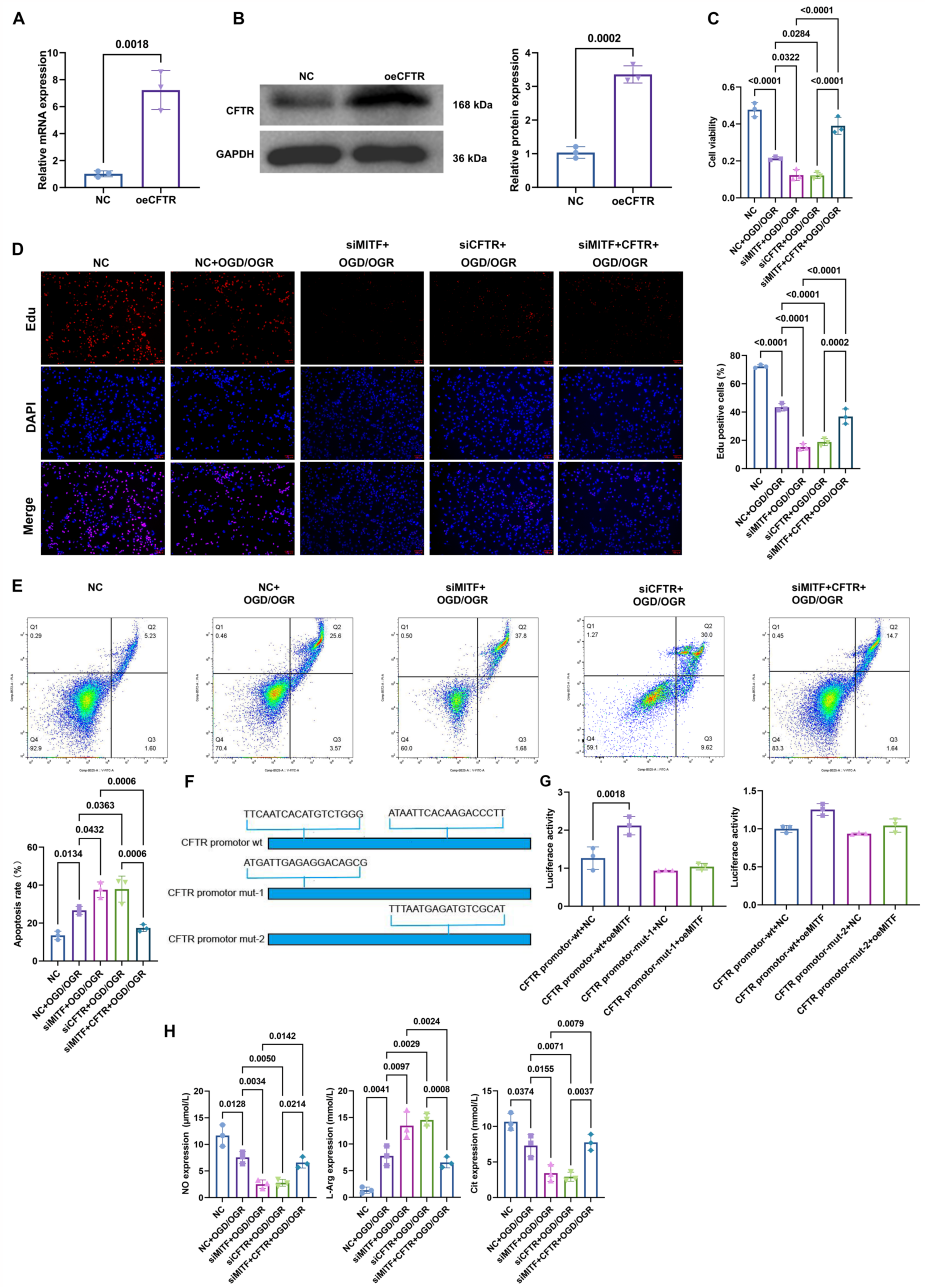
**MITF may alleviate OGD/OGR‐induced injury by regulating CFTR expression.** (A) RT‐qPCR analysis showing successful overexpression of CFTR in AC16 cells transfected with CFTR plasmid (NC vs. oeCFTR). (B) Western blot analysis of CFTR expression after CFTR overexpression. Then NC and NC+OGD/OGR served as baseline and model controls, while siMITF, siCFTR, and siMITF+CFTR were used as loss‐of‐function and rescue controls. (C) Cell viability across experimental groups, with CFTR rescue showing partial improvement. (D) EdU staining indicating restored proliferation after CFTR re‐expression. (E) Apoptosis analysis showing attenuation of MITF knockdown‐induced apoptosis by CFTR restoration. (F) Predicted MITF binding regions within the CFTR promoter. (G) Dual‐luciferase assay showing MITF‐dependent activation of the CFTR promoter. (H) NO, L‐arginine, and citrulline levels measured in AC16 cells under NC, OGD/OGR, siMITF+OGD/OGR, and siMITF+oeCFTR+OGD/OGR conditions. Each experiment included three biological replicates. Data shown as mean ± SD; one‐way ANOVA.

To first confirm the CFTR overexpression model, AC16 cells were transfected with a CFTR plasmid. RT‐qPCR analysis showed a marked increase in CFTR mRNA compared with NC, and Western blot confirmed elevated CFTR protein levels (Figure 6A,B). To further clarify the functional relationship between MITF and CFTR under OGD/OGR conditions, we compared the effects of MITF knockdown, CFTR knockdown, and CFTR rescue across five groups. Compared with the NC group, the NC+OGD/OGR group showed a clear decline in cell viability and proliferation, accompanied by an increase in apoptosis. After MITF knockdown, these changes became more pronounced, with further reductions in viability and EdU‐positive cells, as well as a higher apoptotic rate. A similar pattern was observed in the siCFTR+OGD/OGR group. AC16 cells express detectable levels of endogenous CFTR, which allows us to assess both its reduction after MITF knockdown and its functional involvement under OGD/OGR conditions. Importantly, re‐expression of CFTR in MITF‐silenced cells partially restored cell viability and proliferation and reduced apoptosis relative to the siMITF+OGD/OGR group (Figure 6C–E). Bioinformatic analysis identified two potential MITF‐responsive regions within the CFTR promoter (Figure 6F). Dual‐luciferase assays supported that MITF enhances CFTR promoter activity, and mutation of either predicted binding site reduced this response (Figure 6G). To further investigate whether CFTR contributes to metabolic regulation under OGD/OGR, NO, L‐arginine, and citrulline levels were measured in AC16 cells with different conditions. Compared with NC, NC+OGD/OGR reduced NO and citrulline and increased L‐arginine, while siMITF+OGD/OGR and siCFTR+OGD/OGR further amplified these changes. CFTR overexpression in MITF‐silenced cells partially restored NO and citrulline levels and reduced L‐arginine relative to siMITF+OGD/OGR (Figure 6H). Taken together, these findings suggest that MITF reduction and CFTR suppression have similar effects on OGD/OGR‐induced injury, and restoring CFTR expression can attenuate the functional deficits caused by MITF knockdown. To extend these observations beyond cultured cells, we next examined the MITF‐CFTR axis in a rat I/R model.

### 3.7 CFTR Knockdown Aggravates OGD/OGR‐Induced Injury in AC16 Cells, and ARG1 Inhibition Partially Restores Cell Function

**FIGURE 7:**
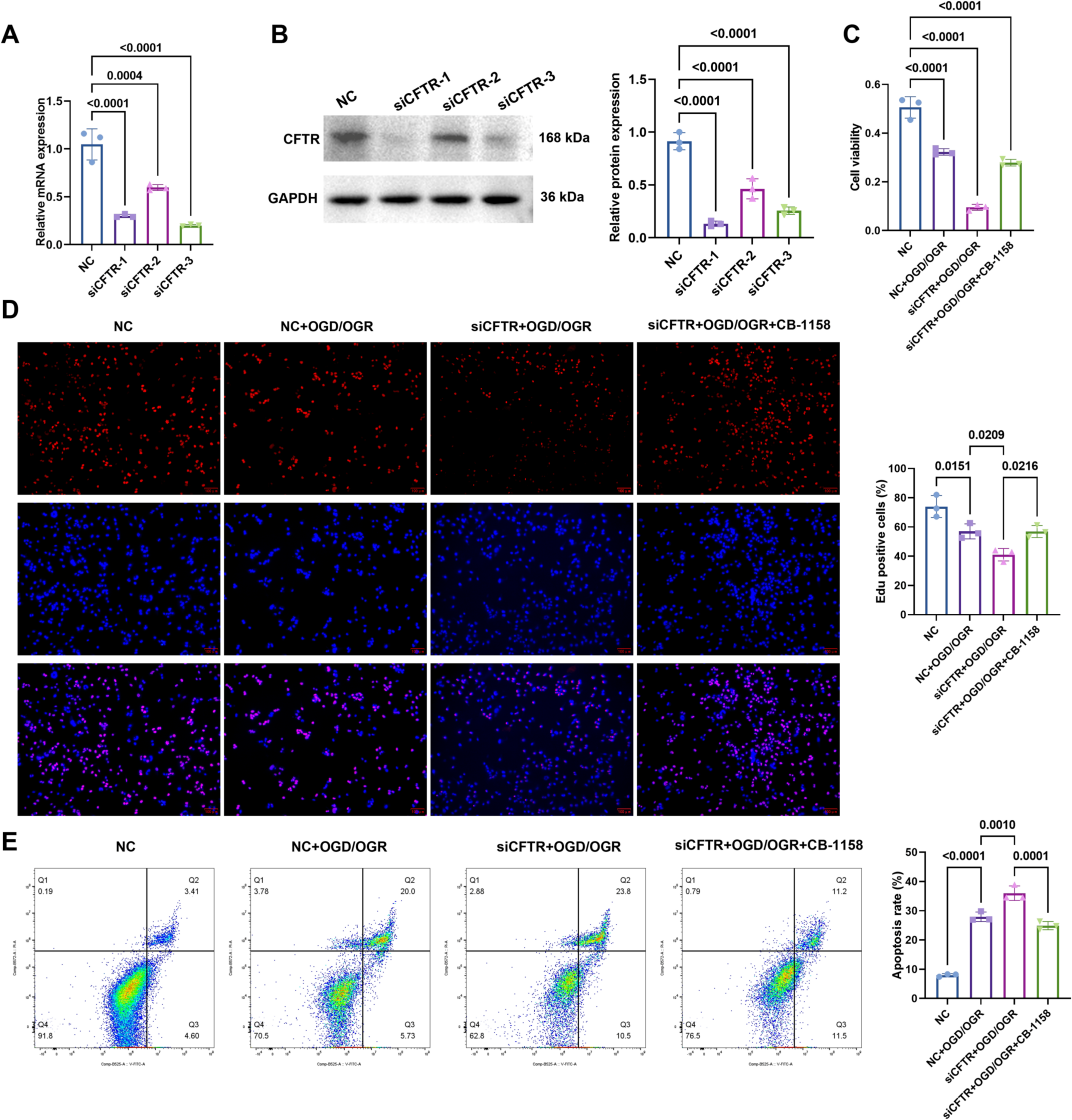
**Effects of CFTR knockdown and ARG1 inhibition on AC16 cells under OGD/OGR conditions.** (A) RT‐qPCR detection of CFTR mRNA expression in AC16 cells transfected with NC or three CFTR‐targeting siRNAs (siCFTR‐1, siCFTR‐2, and siCFTR‐3). (B) Western blot analysis of CFTR protein levels in the same groups. Cell viability assessed by CCK‐8 in NC, NC+OGD/OGR, siCFTR+OGD/OGR, and siCFTR+OGD/OGR+ARG1 inhibitor (CB‐1158) groups. (D) EdU staining for cell proliferation in the same groups. (E) Flow cytometry analysis of apoptosis in the same groups. All experiments included three biological replicates. Data are presented as mean ± SD and analyzed by one‐way ANOVA.

To examine the effect of CFTR knockdown in AC16 cells, we first evaluated three different siRNAs. Compared with NC, all siCFTR groups showed reduced CFTR mRNA levels (Figure 7A) and decreased protein expression (Figure 7B). We next assessed cell function under OGD/OGR conditions. Compared with NC, NC+OGD/OGR cells showed reduced viability and proliferation and increased apoptosis. These changes were further enhanced in the siCFTR+OGD/OGR group. Treatment with the ARG1 inhibitor CB‐1158 in siCFTR+OGD/OGR cells led to increased cell viability and proliferation and decreased apoptosis relative to siCFTR+OGD/OGR without inhibitor (Figure 7C–E). These results indicated that CFTR knockdown is associated with further reductions in viability and proliferation and an increase in apoptosis under OGD/OGR conditions, while ARG1 inhibition partially modulates these effects.

### 3.8 MITF Knockdown or CFTR Silencing Aggravated I/R‐Induced Injury In Vivo, While CFTR Overexpression Appeared to Moderate Several of These Changes

**FIGURE 8:**
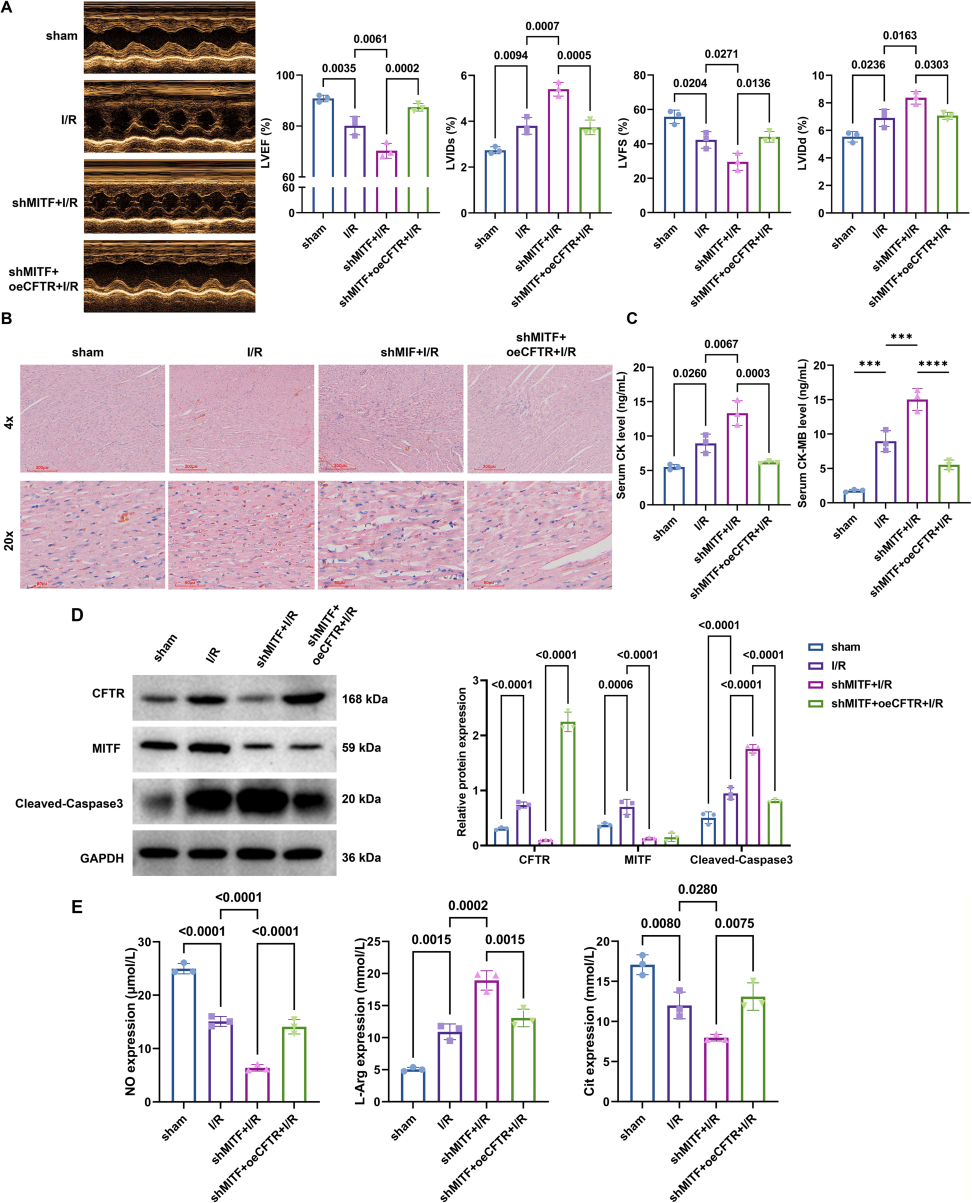
**Effects of MITF knockdown and CFTR overexpression on myocardial I/R injury in rats.** (A) Echocardiographic assessment showing worsened systolic function after MITF knockdown and partial recovery following CFTR overexpression. (B) H&E staining demonstrating increased tissue damage with MITF silencing and improved morphology with CFTR rescue. (C) Elisa analysis of the CK and CK‐MB levels. (D) Western blot analysis of CFTR (The CFTR band shown corresponds to the mature band C), MITF, and cleaved‐caspase‐3 protein levels. (E) NO, L‐arginine, and citrulline levels measured in myocardial tissue of rats under sham, I/R, shMITF+I/R, and shMITF+oeCFTR+I/R conditions. Each group included six rats. Data presented as mean ± SD and analyzed by one‐way ANOVA.

We next evaluated the effects of MITF silencing and CFTR restoration in the rat I/R model. Compared with the sham group, I/R animals showed a decline in cardiac function, reflected by lower LVEF and LVFS and higher LVIDs and LVIDd. These changes were more pronounced in the shMITF+I/R group. In the shMITF+oeCFTR+I/R group, several functional parameters showed partial improvement relative to shMITF+I/R (Figure 8A). Histological analysis supported these findings. Myocardial fibers in the I/R group displayed structural disturbance and inflammatory infiltration. These alterations became more evident after MITF knockdown, whereas CFTR overexpression in MITF‐silenced hearts reduced the extent of tissue disruption (Figure 8B). Serum CK and CK‐MB levels were elevated in the I/R group and further increased after MITF silencing. Animals in the shMITF+oeCFTR+I/R group showed lower CK and CK‐MB concentrations compared with shMITF+I/R (Figure 8C). Protein expression analysis showed a clear change across the four groups. Compared with the sham group, CFTR level increased after I/R, while decreased when MITF was silenced. MITF protein showed a similar trend. Cleaved‐caspase‐3 increased after I/R and rose further with MITF knockdown. When CFTR was re‐expressed in MITF‐silenced rats, CFTR protein increased, and cleaved‐caspase‐3 level reduced compared with the shMITF+I/R group (Figure 8D). Furthermore, NO, L‐arginine, and citrulline levels were measured in myocardial tissue across sham, I/R, shMITF+I/R, and shMITF+oeCFTR+I/R groups. Compared with sham, I/R reduced NO and citrulline and increased L‐arginine, while MITF knockdown further amplified these changes. CFTR overexpression in MITF‐silenced hearts partially restored NO and citrulline levels and reduced L‐arginine relative to shMITF+I/R (Figure 8E). These results indicate that loss of MITF aggravates myocardial injury following I/R, and restoring CFTR expression can ease some of the functional, molecular, and metabolic changes observed in MITF‐deficient hearts.

## 4 Discussion

MIRI remains a major challenge in the management of acute myocardial infarction, largely due to its complex and incompletely defined molecular mechanisms [27]. In this study, we identified MITF as a transcriptional regulator of CFTR through integrated bioinformatics analysis and verified its functional relevance in both OGD/OGR‐treated AC16 cells and the rat ischemia–reperfusion model. Our data show that the MITF‐CFTR axis is altered during I/R injury and that changes in this axis influence cardiomyocyte injury responses. By incorporating both cellular and animal evidence, we observed that MITF reduction aggravated injury in OGD/OGR and I/R models, whereas restoring CFTR expression partially alleviated functional and structural damage. This study provides experimental support that MITF may regulate CFTR expression during MIRI. Several metabolic pathways, including arginine biosynthesis, appeared in our enrichment results and may warrant further study. These signals were first identified by bioinformatic analysis, and our subsequent measurements of NO, L‐arginine, and citrulline provided preliminary experimental support for a possible association, although the underlying mechanism was not directly resolved in this work. The enrichment of arginine biosynthesis in our analysis may reflect a broader metabolic response during reperfusion in which CFTR could participate, although the direct mechanism linking CFTR to this pathway was not fully resolved in our experiments.

Bioinformatics is an important tool for elucidating the molecular mechanisms of complex diseases, with the advantage of efficiently processing and analyzing large amounts of biological data to reveal potential biological mechanisms [28]. In the study of MIRI, bioinformatics has been used to screen for DEGs, construct PPI networks, and identify key transcription factors [29, 30]. In this study, differential expression analysis based on the GSE6381 dataset revealed 215 upregulated and 151 downregulated genes in MIRI tissues, among which the high expression of CFTR was validated in the external dataset GSE249812, and ROC curve analysis showed an area under the curve > 0.60, indicating that CFTR might function as a prospective diagnostic indicator for MIRI. This pattern was further supported in the independent external dataset GSE123342, in which CFTR, PSMC3, AHNAK, and CEMP1 showed an overall upward trend, whereas ARG1 was reduced, strengthening the reproducibility of the bioinformatic findings across datasets.

Beyond its classical role in epithelial tissues, several studies suggest that CFTR also participates in cardiac physiology [31, 32]. In cardiomyocytes, CFTR‐mediated chloride conductance has been implicated in the regulation of membrane ionic homeostasis and action potential duration, and may also affect cell volume and intracellular calcium handling, processes that are highly relevant during ischemia–reperfusion injury [32, 33]. Altered CFTR activity has been linked to disturbed Cl^−^ balance, increased oxidative stress, impaired mitochondrial stability, and suboptimal recovery of contractile function after reperfusion [33]. These cardiac‐specific observations suggest that CFTR may influence myocardial susceptibility to I/R injury. In our study, dynamic changes in CFTR expression in both OGD/OGR‐treated AC16 cells and I/R‐injured rat hearts support this possibility and highlight the importance of exploring its mechanistic role specifically within cardiac tissue. Notably, CFTR is traditionally considered only a chloride channel protein [34], and this study first revealed its transcriptional regulatory mechanisms in cardiac protection, providing new evidence for the functional diversity of ion channel proteins.

KEGG enrichment analysis showed that CFTR‐related genes mapped to several pathways, including arginine biosynthesis. This reflects a computational association. Arginine biosynthesis pathway plays a significant role in cell proliferation, apoptosis, and inflammatory responses [35]. As a precursor for NO synthesis, arginine could activate the cGMP/PKG signaling pathway, inhibit the opening of the mitochondrial permeability transition pore, alleviate calcium overload and oxidative stress, and regulate the restoration of autophagy flux (forming a synergy with the enriched ABC transporters pathway) [36, 37]. Notably, the core gene ARG1 was significantly downregulated in MIRI. As an arginine‐degrading enzyme, ARG1 downregulation may influence substrate availability [38], although the underlying mechanisms remain unclear. At this stage, the relationship between CFTR and arginine‐related metabolism should still be interpreted cautiously. Although enrichment analysis, metabolite measurements, and the partial response to ARG1 inhibition support a possible association, these data do not yet establish a direct regulatory axis between CFTR and arginine biosynthesis.

The transcription factor regulatory network constructed by the KnockTF database showed that CFTR was regulated by multiple stress‐related transcription factors, including TP53 and STAT3, with MITF serving as a core node whose function may intersect with other pathways. For instance, the activation of MITF under hypoxic conditions may involve interaction with HIF‐1α to co‐regulate the expression of downstream target genes. This multidimensional dissection of the regulatory network highlights the irreplaceability of bioinformatics in the study of complex disease mechanisms. MITF is a key transcription factor that is widely involved in biological processes including cell proliferation, differentiation, and apoptosis [39]. MITF has established roles in regulating cell survival pathways (e.g., apoptosis resistance in melanoma) and metabolic reprogramming [40, 41]. However, its potential function in regulating CFTR expression, especially through pathways such as arginine biosynthesis, represents a compelling mechanistic gap. We hypothesize that MITF may ease MIRI by regulating CFTR expression, forming a regulatory axis that influences cell stress responses. Whether metabolic pathways are involved remains to be clarified. Our results shown that CFTR and MITF levels were notably elevated in OGD/OGR models. This expression change may reflect the stress response of cardiomyocytes to I/R injury. As an important ion channel protein, changes in CFTR expression may directly affect the ionic balance and signaling of cardiomyocytes. As a transcription factor, MITF may influence the function and survival of cardiomyocytes by regulating the expression of multiple downstream genes. Functional experiments on cells have shown that knocking down MITF exacerbated OGD/OGR‐induced cardiomyocyte injury (reduced viability and increased apoptosis), while overexpressing CFTR reversed this effect, confirming that CFTR is a key downstream effector molecule of MITF. Conversely, MITF overexpression showed the opposite trend under OGD/OGR conditions, with partial improvement in viability, proliferation, apoptosis, and arginine‐related metabolite changes, which further supports a protective association of MITF in this setting. Consistent with the in vitro findings, MITF silencing in I/R‐treated rats led to poorer cardiac function, higher CK/CK‐MB levels, and more severe histological damage, while CFTR overexpression partially attenuated these changes. These in vivo data strengthen the notion that MITF‐CFTR signaling participates in myocardial injury responses beyond the cellular stress setting. This result revealed the protective role of the MITF‐CFTR axis in maintaining cardiomyocyte survival. Although arginine‐related pathways first emerged from enrichment analysis, the metabolite data and the partial effect of ARG1 inhibition suggest that this pathway may be functionally relevant; however, the direct mechanistic link to CFTR regulation remains unresolved [42]. Meanwhile, as a chloride channel, abnormal expression of CFTR may affect the electrophysiological balance of cardiomyocytes, and the regulatory role of MITF provides new intervention ideas for correcting this imbalance. Dual‐luciferase assays supported that MITF enhanced CFTR promoter activity, whereas ChIP‐qPCR provided direct evidence of MITF enrichment at the CFTR promoter. By contrast, TP53 and STAT3 were predicted in the transcription factor network but did not show detectable enrichment in our ChIP‐qPCR assay, suggesting that MITF may play a more direct role in this regulatory context. This finding expands the known functions of MITF—previous studies have mainly associated MITF with melanogenesis and cell survival [39, 43], and this study is the first to discover its role in MIRI through targeting CFTR. Moreover, this finding broadens the traditional understanding: CFTR, as a chloride channel, has been primarily associated with cystic fibrosis [44], and This study provides evidence that MITF may influence CFTR expression during MIRI and that CFTR modulation is associated with changes in injury‐related readouts.

Although this study has revealed the role of the MITF‐CFTR axis in MIRI through bioinformatics analysis and cell experiments, there are still some limitations. First, the sample size of GSE6381 was small for WGCNA, so the network results should be considered exploratory and interpreted with caution. Second, although the function of the MITF‐CFTR axis has been preliminarily supported by our rat I/R experiments, more in vivo evidence is still needed to strengthen the causal relationship. In the future, it will be necessary to construct mice with cardiac‐specific MITF knockout to observe phenotypic changes in in vivo MIRI models. Second, the specific mechanisms of action of the arginine biosynthesis pathway have not yet been fully elucidated. Although changes in NO, L‐arginine, and citrulline, together with the partial effect of ARG1 inhibition, provided preliminary support for arginine‐related involvement, metabolomics analysis and more direct mechanistic experiments are still required to determine whether CFTR regulation directly affects arginine metabolism and NO production. Although TP53 and STAT3 were also identified as candidate CFTR‐related transcription factors in the bioinformatic analysis, their relationship with MITF was not explored further in this study. Whether these factors interact with MITF or act together under OGD/OGR conditions remains unclear and will need to be addressed in future experiments, such as Co‐IP or more detailed promoter‐binding analyses. Additionally, whether the regulation of the MITF‐CFTR axis is universally present in human MIRI samples still needs to be verified in larger‐scale clinical cohorts. In addition, the public datasets used in this study did not provide sufficiently detailed and matched clinical outcome information, so the potential prognostic value of MITF and CFTR could not be evaluated in the present work. It is worth noting that MITF has oncogenic effects in diseases such as melanoma [45], but its function in the heart may be tissue‐specific. Therefore, the development of heart‐targeted MITF activators or CFTR modulators will require strict assessment of their safety and side effects. Future research could combine single‐cell sequencing technology to dissect the expression differences of MITF in different cardiomyocyte subpopulations and further refine its mechanisms of action. In addition, the functional experiments were conducted in AC16 cells, and validation in primary cardiomyocytes would further enhance physiological relevance; this will be incorporated into subsequent studies.

## 5 Conclusion

In summary, this study combined bioinformatics, cell experiments, and in vivo evidence to demonstrate that MITF directly regulates CFTR expression and participates in the cellular response to I/R injury. MITF knockdown aggravated both OGD/OGR‐induced and I/R‐induced injury, while CFTR restoration partially reversed these effects. MITF overexpression showed the opposite trend in AC16 cells, and arginine‐related metabolite changes were observed in both cellular and animal models, although their mechanistic connection to MITF‐CFTR regulation requires further study. These findings suggest that the MITF‐CFTR axis contributes to myocardial injury processes and may provide a promising direction for future therapeutic exploration, although further mechanistic studies are still required.

## Author Contributions

Baoxin Tang, Chenying Zhu, Heqing Wang, and Mingkui Gao: data curation, investigation, formal analysis, resources, and writing – review and editing.

Tieyan Li: conceptualization, methodology, writing – original draft, and writing – review and editing.

## Funding

This work was supported by the Academic Leaders Training Program of Pudong Health Bureau of Shanghai (Grant No. PWRd2024‐14) received by Tieyan Li.

## Disclosure

An AI‐powered language assistant (Kimi K2.6, Moonshot AI, accessed July 9, 2026) was used for spell‐checking and verification of technical terminology in the manuscript. The tool was applied to the full text of the manuscript. All research design, data collection, data analysis, interpretation of results, and drafting of scientific arguments were conducted independently by the authors. AI outputs were verified against standard references and the authors' domain expertise. No sensitive, proprietary, or human subject data were entered into the AI system.

## Ethics Statements

Animal experiments were performed in this study and were approved by the Guangzhou Forevergen Biosciences Animal Experimentation Ethics Committee (Approval Number: IACUC‐AEWC‐F250912002). Publicly available human transcriptomic datasets from the GEO database were analyzed, and no newly collected human samples or identifiable personal data were involved.

## Supporting information


**Figure S1:** **External validation of core genes in the GSE123342 dataset.** (A) Heatmap showing the expression of seven core genes in peripheral blood samples from 67 acute myocardial infarction (control) and 66 myocardial ischemia/reperfusion (MIR) samples. (B) Boxplots showing relative expression levels of the seven core genes between control and MIR groups. (C) ROC curves of the seven core genes, with corresponding area under the curve (AUC) values.

**Figure S2:** **MITF overexpression protects AC16 cells against OGD/OGR‐induced injury.** (A) RT‐qPCR measurement of MITF mRNA in AC16 cells transfected with control plasmid (oeNC) or MITF overexpression plasmid (oeMITF). B. Western blot analysis of MITF protein levels in oeNC and oeMITF groups. (C) Cell viability measured by CCK‐8 in NC, oeMITF, NC+OGD/OGR, and oeMITF+OGD/OGR groups. (D) EdU staining to assess cell proliferation in the same groups. (E) Flow cytometry analysis of apoptosis. (F) Measurement of NO, L‐arginine, and citrulline levels in the same groups using commercial kits. Experiments were performed in three biological replicates. Data are shown as mean ± SD and analyzed by one‐way ANOVA.

## Data Availability

The original contributions presented in the study are included in the article; further inquiries can be directed to the corresponding author.
