# Supplementary material for: MITF Regulates CFTR Expression to Participate in Myocardial Ischemia–Reperfusion Injury
Source: J Gene Med. 2026 Aug 6;28(8):e70102. doi: 10.1002/jgm.70102 (PMC13447130; doi:10.1002/jgm.70102)
Supplement: Supplementary file 1 [file JGM-28-e70102-s001.pdf]

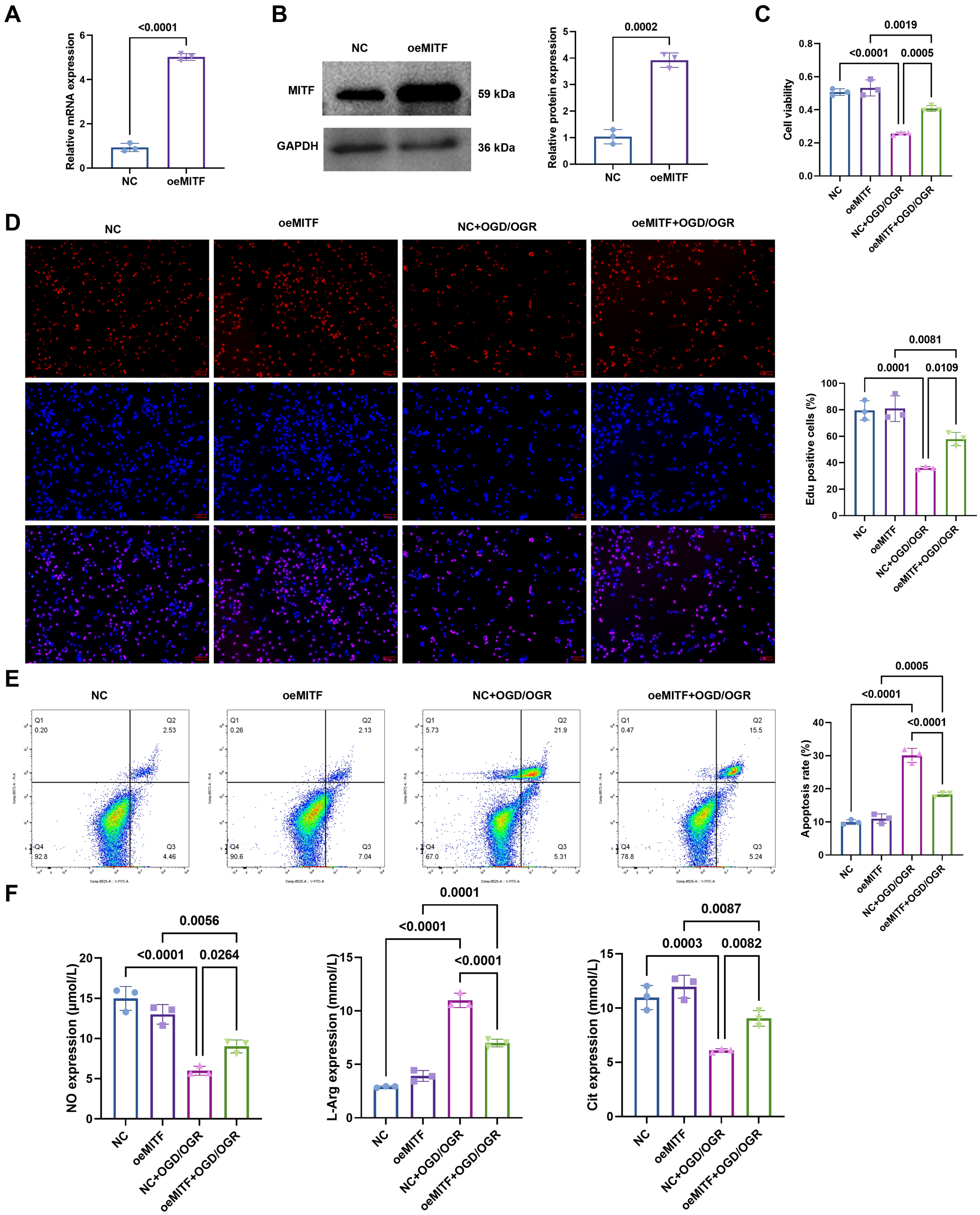
A
Relative mRNA expression
<0.0001
NC
oeMITF
B
NC
oeMITF
MITF
59 kDa
GAPDH
36 kDa
Relative protein expression
0.0002
NC
oeMITF
C
Cell viability
0.0019
<0.0001
0.0005
NC
oeMITF
NC+OGD/OGR
oeMITF+OGD/OGR
D
NC
oeMITF
NC+OGD/OGR
oeMITF+OGD/OGR
Edu positive cells (%)
0.0081
0.0001
0.0109
NC
oeMITF
NC+OGD/OGR
oeMITF+OGD/OGR
E
NC
oeMITF
NC+OGD/OGR
oeMITF+OGD/OGR
Q1 0.20
Q2 2.53
Q4 92.8
Q3 4.46
Q1 0.26
Q2 2.13
Q4 90.6
Q3 7.04
Q1 5.73
Q2 21.9
Q4 67.0
Q3 5.31
Q1 0.47
Q2 15.5
Q4 78.8
Q3 5.24
Apoptosis rate (%)
0.0005
<0.0001
<0.0001
NC
oeMITF
NC+OGD/OGR
oeMITF+OGD/OGR
F
NO expression (μmol/L)
0.0056
<0.0001
0.0264
L-Arg expression (mmol/L)
0.0001
<0.0001
<0.0001
Cit expression (mmol/L)
0.0087
0.0003
0.0082
NC
oeMITF
NC+OGD/OGR
oeMITF+OGD/OGR
