# Supplementary material for: MITF Regulates CFTR Expression to Participate in Myocardial Ischemia–Reperfusion Injury
Source: J Gene Med. 2026 Aug 6;28(8):e70102. doi: 10.1002/jgm.70102 (PMC13447130; doi:10.1002/jgm.70102)
Supplement: Supplementary file 2 [file JGM-28-e70102-s002.pdf]

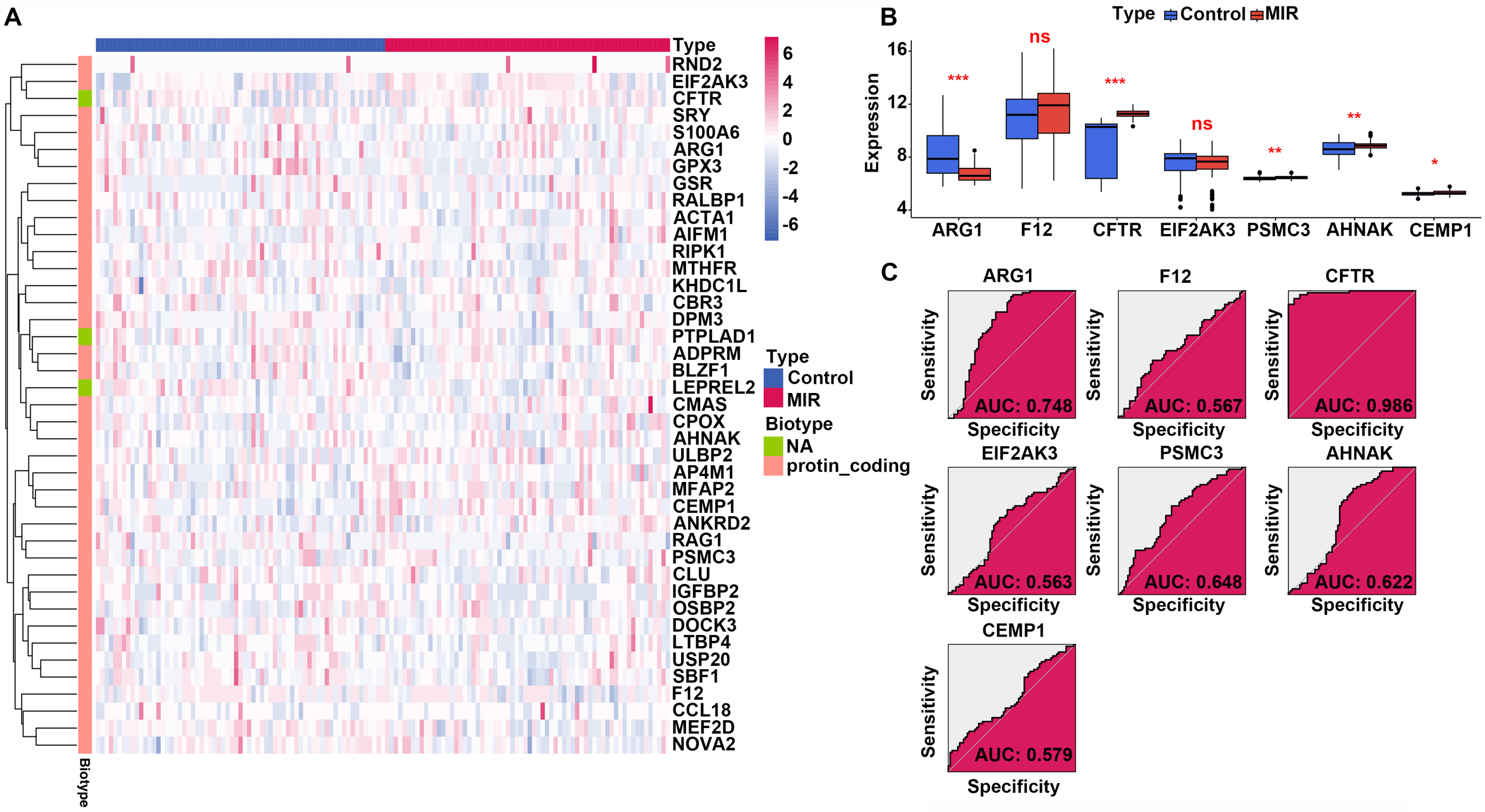

A
Type
RND2
EIF2AK3
CFTR
SRY
S100A6
ARG1
GPX3
GSR
RALBP1
ACTA1
AIFM1
RIPK1
MTHFR
KHDC1L
CBR3
DPM3
PTPLAD1
ADPRM
BLZF1
LEPREL2
CMAS
CPOX
AHNAK
ULBP2
AP4M1
MFAP2
CEMP1
ANKRD2
RAG1
PSMC3
CLU
IGFBP2
OSBP2
DOCK3
LTBP4
USP20
SBF1
F12
CCL18
MEF2D
NOVA2
Biotype
6
4
2
0
-2
-4
-6
Type
Control
MIR
Biotype
NA
protin_coding
B
Type Control MIR
Expression
16
12
8
4
***
ns
***
ns
**
**
*
ARG1
F12
CFTR
EIF2AK3
PSMC3
AHNAK
CEMP1
C
ARG1
AUC: 0.748
F12
AUC: 0.567
CFTR
AUC: 0.986
EIF2AK3
AUC: 0.563
PSMC3
AUC: 0.648
AHNAK
AUC: 0.622
CEMP1
AUC: 0.579
Sensitivity
Specificity
